# Modulation of T helper 1 and T helper 2 immune balance in a murine stress model during *Chlamydia muridarum* genital infection

**DOI:** 10.1371/journal.pone.0226539

**Published:** 2020-05-15

**Authors:** Tesfaye Belay, Elisha Martin, Gezelle Brown, Raenel Crenshaw, Julia Street, Ashleigh Freeman, Shane Musick, Tyler J. Kinder

**Affiliations:** Bluefield State College, Bluefield, WV, United States of America; Alabama State University, UNITED STATES

## Abstract

A murine model to study the effect of cold-induced stress (CIS) on *Chlamydia muridarum* genital infection and immune response has been developed in our laboratory. Previous results in the lab show that CIS increases the intensity of chlamydia genital infection, but little is known about the effects and mechanisms of CIS on the differentiation and activities of CD4+ T cell subpopulations and bone marrow-derived dendritic cells (BMDCs). The factors that regulate the production of T helper 1 (Th1) or T helper 2 (Th2) cytokines are not well defined. In this study, we examined whether CIS modulates the expressions of beta-adrenergic receptor (β-AR), transcription factors, hallmark cytokines of Th1 and Th2, and differentiation of BMDCs during *C*. *muridarum* genital infection in the murine model. Our results show that the mRNA level of the beta2-adrenergic receptor (β2-AR) compared to β1-AR and β3-AR was high in the mixed populations of CD4+ T cells and BMDCs. Furthermore, we observed decreased expression of T-bet, low level of Interferon-gamma (IFN-γ) production, increased expression of GATA-3, and Interleukin-4 (IL-4) production in CD4+ T cells of stressed mice. Exposure of BMDCs to Fenoterol, β2-AR agonist, or ICI118,551, β2-AR antagonist, revealed significant β2-AR stimulation or inhibition, respectively, in stressed mice. Moreover, co-culturing of mature BMDCs and naïve CD4+ T cells increased the production of IL-4, IL-10, L-17, and IL-23 cytokines, suggesting that stimulation of β2-AR leads to the increased production of Th2 cytokines. Overall, our results show for the first time that CIS promotes the switching from a Th1 to Th2 cytokine environment. This was evidenced in the murine stress model by the overexpression of GATA-3 concurrent with elevated IL-4 production, reduced T-bet expression, and IFN-γ secretion.

## Introduction

Chlamydia genital infection caused by *Chlamydia trachomatis* is the most common bacterial sexually transmitted disease worldwide [1]. This infection, if left untreated, leads to the development of pelvic inflammatory disease (PID), fallopian tube scarring, ectopic pregnancy, infertility, and neonatal conjunctivitis [2,3]. Epidemiologic data from the Centers for Disease Control and Prevention and World Health Organization indicate that more than 90 million new cases occur annually worldwide, with approximately 4 million of those in the United States [4]. Chlamydia genital infection disproportionately affects populations of low socioeconomic status, and more particularly, the African-American population [5,6]. The reasons are not well known, but increased stress associated with low socioeconomic conditions may have a major role in the persistently high rate of the disease [7].

Several studies in animal models have demonstrated that anti-chlamydial T cell responses in the local genital mucosa play a significant role in the clearance of *C*. *trachomatis* from the genital tract [8–12]. It is known that T cells mediate immunity to *C*. *trachomatis*, specifically T-helper 1 (Th1) cells, through IFN-*γ*-dependent and -independent pathways [13–16]. Moreover, other studies in human subjects [17,18] and animal models [19, 20] have exhibited vital aspects of genital chlamydial infection, including similar course of active infection, pathologic consequences, or sequelae of infection, tubal inflammation, hydrosalpinx formation, and infertility. It is well recognized that repeated chlamydia genital infection in animal models [21–24] and human subjects [25,26] is known to have a much higher risk of a tubal obstruction than those with less exposure to *C*. *trachomatis*.

*Chlamydia muridarum*, previously known as the mouse pneumonitis strain of *C*. *trachomatis*, is commonly used in the study of chlamydia immunity, pathogenesis, and vaccine development [27–29]. Infectivity, elicitation of pathologies, and immunological findings in *C*. *muridarum* murine models are not vastly different from those that occur with *C*. *trachomatis* infections in humans [30–33].

Psychological or physical stress resulting from the hardship of life in society has significant impacts on public health [34–38]. Two stress hormones, glucocorticoids, and catecholamines serve as the major mediators of stress responses, ultimately resulting in either immunosuppression or immunostimulation in the host [39–41]. Norepinephrine (NE) is one of the catecholamines released during stressful conditions that bind and stimulates the β-AR subtypes, which are predominantly expressed on immune cells [42–44]. Application of cold water as a stressor in animal models, including mice, has resulted in changes in immune responses that correlate with the activity of the neuroendocrine system of corticosteroids and catecholamines [45–48].

Although stress is implicated as a risk factor for various infections, its effect on chlamydia genital infection is unknown. We have previously shown that cold-water stress induces the production of catecholamines, which may play a critical role in the modulation of the immune system, thus leading to increased intensity of *C*. *muridarum* genital infection [49]. We also have shown that supplementation of NE to splenic T cells *in vitro* exerts an immunosuppressive effect on cytokine production, which is associated with decreased C. *muridarum* shedding in the genital tract of *β*1Adr/*β*2-AR knockout mice. Furthermore, a severe pathology with a higher rate of infertility was observed in the *C*. *muridarum* infected stressed mice as opposed to infected non-stressed mice [50]. However, our understanding of the expression and function of Th1 and Th2 during CIS remains limited.

In this study, we have sought to determine whether cold-stress treatment of mice could lead to imbalanced Th subsets during *C*. *muridarum* genital infection. We hypothesized that cold-stress treatment leads to the skewing of CD4+ Th1 to Th2 cell production. Our method of measuring this skew was to determine mRNA levels of T-bet and GATA-3 and signature cytokines produced by CD4+ T cells during *in vitro* proliferation. Moreover, we evaluated the differentiation and cytokine production ability of BMDCs and its influence on the induction of Th cells upon co-culturing *in vitro*.

## Materials and methods

### *Chlamydia* stock culture and McCoy cell line

McCoy murine cell line and stock culture of *Chlamydia muridarum*, previously known as *Chlamydia trachomatis* agent of mouse pneumonitis, were kindly provided by Dr. Joseph Igietseme, then at Morehouse School of Medicine and currently at CDC, Atlanta, GA.

### Animals

Six- to seven-week-old female BALB/c strain mice were purchased from Hilltop Lab Animals, Inc. (Scottsdale, PA). Mice were housed in a vivarium at Bluefield State College (BSC) located in the Basic Science Building of the School of Arts and Sciences. Mice were given food and water *ad libitum* in an environmentally controlled room with equal daylight and night hours. All experimental animal protocols in this study were carried out in strict accordance with the recommendations in the Guide for the Care and Use of Laboratory Animals of the National Institutes of Health. The protocol was approved by the BSC Institutional Animal Care and Use Committee prior to the beginning of the research.

### Cold-water stressing protocol

To relieve the stress of transport, mice were acclimated for 7 days before prior to experimentation. Mice were then divided into three groups: stressed, handled/non-stressed, and not handled/non-stressed. To induce chronic stress, five mice at a time were placed in a packet filled with four cm of cold water (4 to 5°C) for five minutes daily for 24 days, as previously described [49,50]. The water level was deep enough to cover the backs of the mice and force them to swim in the cold water. At the end of each stressing period, mice were dried with towels to avoid hypothermia. Hereafter this group is referred to as a “stressed” group. To ensure that the stress was caused by the water treatment and not merely by handling the same protocol with the exception that the water was room temperature (22°C) was applied to a second group of mice. A third group of mice was kept at room temperature without water treatment. Initial data on stress hormone production and *C*. *muridarum* course of infection showed no significant difference between the handled/non-stressed and the not-handled/non-stressed groups. For simplicity, the non-stressed group was chosen as the control throughout the study, and further referred to as the non-stressed group.

### Inoculation and isolation of *C. muridarum* from genital tract swabs

To regulate estrous cycles, all mice were injected subcutaneously with 2.5 mg of progesterone in 100 μL of phosphate-buffered saline (PBS) on day 17 of the 24-day stressing period. On day 24, stressed and non-stressed mice were inoculated intravaginally with 10^5^ IFU of *C*. *muridarum* in a volume of 30 μL of PBS while under Ketamine-Xylazine induced anesthesia to minimize discomfort. The course of infection was monitored by cervicovaginal swabbing at 3-day intervals for the first 42 days of the primary course of infection. C*hlamydia muridarum* was recovered from swabs by staining infected monolayers of McCoy tissue culture with fluorescein isothiocyanate labeled genus-specific anti-chlamydial antibodies purchased from Fisher Scientific (Pittsburgh, PA). Inclusion bodies in 10 fields/well were visualized, imaged, and counted under a fluorescent Mitic AE31E microscope (Carlsbad, CA) and inclusion-forming units per mL (IFU/mL) was determined as previously described in our lab [49].

### Purification of CD4+ T cells from spleen and genital tract of mice and proliferation *in vitro*

Spleens were removed aseptically from each mouse, then minced and teased with forceps in RPMI 1640 complete medium supplemented with 10% fetal bovine serum 1% penicillin/streptomycin, and 0.1% gentamicin (Sigma, St. Louis, MO). Spleen cell suspensions were pressed through a 70-μm cell strainer (Becton Dickinson, Franklin Lakes, NJ) to remove tissue debris. Splenocyte CD4+ T cells were isolated using EasyStep murine T cell enrichment kits based on immunomagnetic negative selection, (STEMCELL Technologies, Vancouver, Canada) following the manufacturer’s instructions. Briefly, the suspension of spleen cells was incubated with the EasySep Ab mixtures to no-CD4+ T cells, followed by the addition of biotin selection mixture and magnetic beads to isolate CD4+ T cells and remove unwanted cells. Genital tract lysates were produced by homogenization and lysates were pressed through a 70-μm cell strainer to purify CD4+ T cells as described above.

Antigen Presenting Cells *(*APCs) from splenocytes of non-stressed mice were prepared by treatment with Mitomycin C (Sigma-Aldrich, St. Louis, MO). Briefly, splenocyte cells were washed by centrifugation at 300 x g for 10 minutes. Freshly prepared Mitomycin C at the concentration of 25 μg/mL was added to 10^7^ splenocyte cells/mL and incubated at 37°C for 30 minutes. Cells were washed four times as above, and a final cell count of 5 x 10^5^ Mitomycin C-treated cells were prepared for proliferation assay.

CD4+ T cell proliferation *in vitro* was performed as described previously [49] with some modifications. Briefly, cells were seeded in triplicate at a density of 5 × 10^5^ CD4+ T cells mixed with 5 x 10^5^ Mitomycin C treated cells per well in a 96-well culture plate (Becton Dickinson). The cells were stimulated with 2.5 μg/mL of concanavalin A (Con A) and cultured for 72 h in a water-jacketed incubator at 37°C and 5% CO_2_ (NuAire, Plymouth, MN). Culture supernatants were collected after 72 h of proliferation then stored at -80°C in preparation for measuring cytokine production using ELISA.

#### Testing the action of NE, Fenoterol or ICI 118,551 on CD4+T cell proliferation

We investigated the influence of synthetic NE, Fenoterol, or ICI 118,55 1 (Sigma-Aldrich, St. Louis, MO) on CD4+T cell cytokine production. Spleen samples were harvested as described above from stressed-infected (SI), non-stressed-infected (NSI), and non-stressed-non-infected (NSNI) mice and isolated using negative selection following the manufacturer’s instructions as described above. Purified T cells were seeded at a density of 5 x 10^5^ CD4+ T cells per well in 96-well plates along with the same number of APCs were exposed to NE (1 μM), Fenoterol (1 μM) and ICI 118,551 (1 μM) for 1 h. Cells were cultured for 72 h at 37° C in the presence/absence of Con A (2.5 μg/mL), (0.3 μg/mL), and irradiated chlamydia antigen. Culture supernatant was collected after 72 h of proliferation and immediately stored at -20°C to be tested for the production of selected cytokines by ELISA.

#### Isolation and generation of bone marrow-derived dendritic cells (BMDCs)

To collect bone marrow cells, the femur and tibia bones were pulverized using a mortar and pestle, and debris was removed by passing the suspension through a 70 μm mesh nylon strainer. Cells were pipetted vigorously up and down to obtain a single-cell suspension, then washed once with complete RPMI 1640, and red blood cells were lysed using cell lysis buffer from Sigma. Purified cells were cultured in a tissue culture flask containing 20 mL RPMI 1640 supplemented with recombinant murine Granulocyte-Macrophage Colony Stimulating Factor (GM-CSF) (10 ng/mL) from BioSource, Invitrogen Cytokines & Signaling (Camarillo, CA). At day 3 of the culture, half of the old culture was replaced with 10 mL of complete RPMI 1640 media and GM-CSF (10 ng/mL). At day 8, all floating cells and loosely adherent cells were collected as immature BMDCs. All cells were counted in Trypan blue, and their viability was confirmed to be over 80%.

#### Cytokine production and Dendritic Cell (DC) maturation upon lipopolysaccharide (LPS) stimulation

To examine the effect of β2-AR activation, immature dendritic cell culture was pre-exposed to NE (1 μM), Fenoterol (1μM) and ICI 118,551(1 μM) for 1 h before stimulation with LPS (5 μg/mL) (Sigma-Aldrich, St. Louis, MO) for 24 h. Supernatants were collected, and the production of different cytokines was assessed using an ELISA kit. To examine the effect of BMDCs on CD4+ T cells proliferation, freshly isolated CD4+ T cells isolated from stressed and non-stressed *C*. *muridarum*-infected mice were co-cultured with matured BMDCs for four days in 96-well plates at a BMDCs: T cells ratio of 1:10. Culture supernatant was collected at day four of co-culturing, and cytokine production was determined by ELISA.

#### Priming Th1 or Th2 cells *in vitro* under polarizing conditions

Naïve CD4 + T cells from stressed and non-stressed groups of mice were isolated using the murine naïve CD4+ T cell isolation kit for activation with the polarizing milieu. Plates coated with 1 μg/mL anti-CD3 from BD PharMingen Inc (San Jose, CA) for overnight were used for stimulation. For Th1 polarization, splenic naïve CD4+ T cells were seeded at 1 x 10^5^ cells/well in the plate and incubated at 37°C for 72 h in the presence of recombinant murine IFN-γ (10 ng/mL) recombinant murine IL-12 (10 ng/mL) and anti-murine IL-4 (100 ng/mL). For Th2 polarization, CD4+ T cells were seeded at 1 x 10^5^ cells/well in the plate and incubated at 37°C for 72 h in the presence of recombinant murine recombinant IL-4 (100 ng/mL) anti-murine IFN-γ (10 ng/mL) and anti-murine IL-12 (10 ng/mL) for Th2 polarization. The CD4+ T cells were stimulated again for another four days in coated plates with fresh medium addition. Culture supernatants were collected to determine the polarization of IL-4 and IFN-γ in stressed and non-stressed CD4+ T cell samples as determined by ELISA.

### Cytokine measurement using ELISA

The level of cytokines in supernatants collected from *in vitro* proliferating CD4+ T cells and BMDCs of different treatment groups was measured using an ELISA kit (Invitrogen, Camarillo, CA) following the manufacturer’s instructions as previously described [50]. Briefly, standard samples and test samples were diluted and added to wells in duplicate and incubated as directed. Optical densities were measured at 450 nm with an ELISA plate reader from Biotech (Winooski, VT). The concentration of the cytokine in each sample was obtained by extrapolation from the standard calibration curve generated in each assay.

#### RNA isolation, cDNA synthesis and Polymerase Chain Reaction (PCR) analysis

Total RNA was isolated from cultured splenocyte and genital tract T cells and BMDCs using the FastPrep24™ or FastPrep® FP120 Instrument, and Fast RNA® Pro Green Kit from MP Biomedicals (Solon, OH) following the manufacturer’s instructions. Briefly, total RNA was released into the protective RNApro™ solution by homogenizing samples in 2 mL tubes using Lysing Matrix D. Total RNA Extraction was completed using chloroform and precipitation. Then the isolated RNA was quantified using a Theme Scientific Nanodrop from Thermo Fisher Scientific (Ashville, NC). RNA sample values ranging from 1.9 to 2.1 at 260/280 and > 2.0 at 260/230 ratio were used for cDNA synthesis.

Oligo primers of target molecules for mRNA determination were purchased from Integrated DNA Technologies (Skokie, IL). The first-strand cDNA for gene expression analysis using real-time qPCR was carried out using the iScript cDNA synthesis kit from Bio-Rad (Hercules, CA) following the manufacturer’s instructions. Briefly, cDNA synthesis was synthesized by mixing 5x iScript reaction iScript reverse transcriptase, and total RNA was mixed in clean Eppendorf tubes then brought to a total of 20 μL using RNase/DNase-free water. The tubes were then incubated at 25°C for 5 minutes, 30 minutes at 42°C, and 5 minutes at 85°C to stop the reaction. qPCR was conducted following the manufacturer’s instructions.

The following Sequence of oligo primers were used in the qPCR assays.

**T-bet** 5’-TCAACCAGCACCAGACAGAG-3’ (forward:), 5’-AACATCCTGTAATGGCTTGTG-3’(reverse);

**GATA-3**-\5’- CTTATCAAGCCCAAGCGAAG-3’(forward), 5-CCCATTAGCGTTCCTCCTC-3’(reverse);

**β1AR**-: 5’-AAACTCTGGTAGCGAAAGGGGAC-3’ (forward), 5’ TCTGCTCATCGTGGTGGGTAAC 3’(reverse);

**β2AR**-5’-AGCCGTTCCCATAGGTTTCG-3’(forward), 5’-CGTCCTCGATTGTGTCTTTCTACG-3’(reverse);

**β3AR**-5’-CGAAGAGCATCACAAGGAGGG-3’ (forward), 5’-CGAAACTGGTTGCGGAACTGTGT-3’(reverse);

**IFN-gamma**-5’- GCCATCAGCAACAACATAAGC-3’(forward), 5’TGAGCTCATTGAATGCTTGG-3’ (reverse);

**IL-4**–5’-CATCGGCATTTTGAACGAG-3’(forward), 5’-CGAGCTCACTCTCTGTGGTG-3’(reverse).

**GAPDH**-5’ CATCACTGCCACCCAGAAGACTG-3’ (forward), 5’-ATGCCAGTGAGCTTCCCGTTCAG-3(reverse)’

Quantitative PCR was performed using BIO-RAD iTaq™ Universal SYBR® Green Supermix following the manufacturer’s instructions on a CFX96 Real-Time System and BioRad CFX Manager. Briefly, iTaq Universal SYBR Green Supermix, forward and reverse primer mix for each gene, RNase/DNase free water, and cDNA samples were added to PCR strips in duplicates. qPCR reactions parameters were incubation at +95°C for 10 s, and thereafter 40 cycles of denaturation at +95°C for 15 s and annealing and extension at + 60°C for 30 s. The ΔΔCT method was used to calculate relative changes in gene expression of target genes determined. Gene expression of stressed and non-stressed mice samples was normalized to the internal control gene, Glyceraldehyde 3-phosphate dehydrogenase (GAPDH).

#### Western blotting of analysis

Protein from murine genital tract lysate, CD4+ T cells, or BMDCs were isolated using the FastPrep-24™ or FastPrep® FP120 Instrument and FastRNA® Pro Green Kit from MP Biomedicals following the manufacturer’s instructions. Antibodies β-Actin (8H10D10) Murine mAb #3700, GATA-3 (D13C9) XP®Rabbit mAb (Biotinylated) #45905, Stat4 (2A2) Murine mAb #5097, and Stat6 (D3H4) Rabbit mAb #5397 were purchased from Cell Signaling Technologies. Anti-β-AR antibody (ab176490) was purchased from Abcam (Cambridge, MA). Purified protein was denatured and separated by sodium dodecyl sulfate-polyacrylamide gel electrophoresis in a Criterion^TM^ Cell (Bio-Rad). Protein bands were transferred to a nitrocellulose membrane in a Criterion^TM^ Blotter (Bio-Rad) following the manufacturer’s instructions. Band formation was detected via an Amplified Opti-4CN Substrate Kit following the manufacturer’s instructions. Relative protein expression or densitometry was calculated using the Bio-Rad ChemiDOC molecular imager and software following the manufacturer’s instructions. Equivalent protein loading and transfer efficiency were verified by using β–actin as a control.

#### Establishing interaction networks of gene and proteins

Cytoscape is a social network of open source software that is used to visualize molecular interaction networks and biological pathways. Data on gene expression and cytokine production in stressed and non-stressed mice with or without *C*. *muridarum* genital infection were collected for interaction with other important genes or proteins extracted from the database. Related protein networks of up-regulated or down-regulated genes or proteins were visualized using the cystoscope database and software downloaded from Cytoscape.org.

### Statistical analysis

All data are expressed as mean+/- standard errors of the mean (SEM) unless otherwise stated. Statistical significance between any two groups was tested using Student’s *t*-test, and ANOVA was used to test a statistical difference between more than two groups. Statistical significance was at the level of *P < 0*.*05*.

## Results

### Cold water-induced stress increases the intensity of *C*. *muridarum* genital infection during primary infection

**Fig 1:**
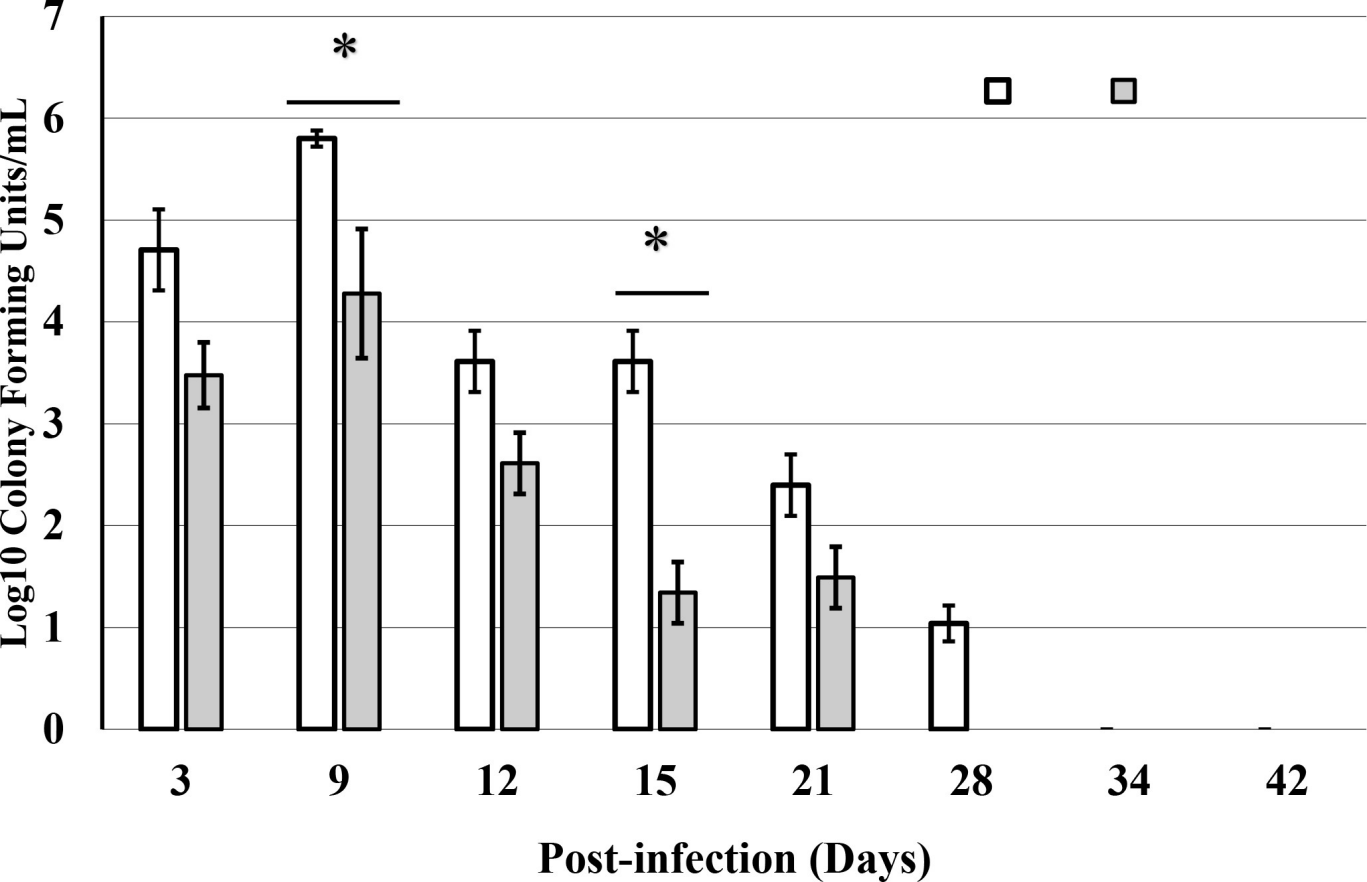
Course of *C*. *muridarum* genital infection in stressed and non-stressed mice as measured by quantitation of viable organisms shed from the genital tract. Each data is mean +/_ SEM of inclusion forming units/milliliter determined from McCoy cell culture representing combined results of two separate experiments (n = 5 mice per group). A statistical difference in counts was observed between stressed and non-stressed mice on day 9 and 15 after infection (p <0.05). Shedding of C. *muridarum* in non-stress mice or stressed mice after day 28, indicating the resolution of infection.

To determine the level of infection in stressed and non-stressed mice along with immune response analysis, we measured the number of viable *C*. *muridarum* present on vaginal swabs collected every three days post-infection. As shown in **Fig 1**, there are higher numbers of chlamydia inclusion in stressed mice compared to non-stressed mice, indicating that CIS increases the intensity of *C*. *muridarum* genital infection. Our results are consistent with our previous findings of increased *C*. *muridarum* shedding in regions of the genital tract of stressed mice compared to non-stressed mice [49,50].

### Cold-induced stress results in differential gene expression of the β-AR subtypes in murine genital tract CD4+ T cells during *C*. *muridarum* infection

**Fig 2:**
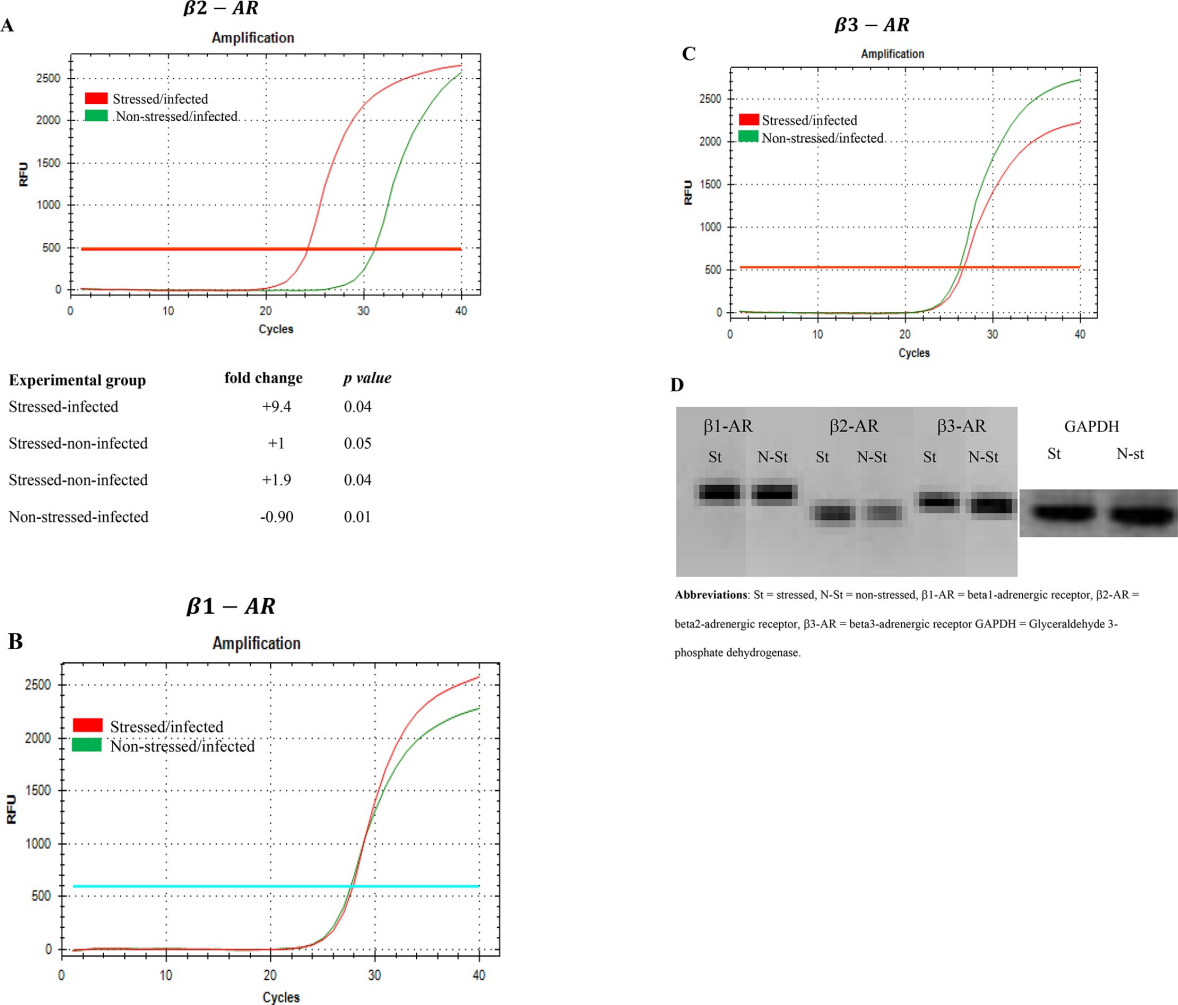
Gene expression profiles of β-AR) subtypes in CD4+ T cells isolated from the genital tract of stressed and non-stressed mice during *C*. *muridarum* genital infection. Amplification cures and fold-changes of mRNA levels of β2-AR subsets normalized to the housekeeping gene, GADPH, is shown. **A**) β2-AR, **B**) β1 –AR, **C**) β3-AR, D) qPCR products on gel electrophoresis. ***Abbs***: **St** = stressed, **Non-St** = none-stressed. Data shown are representative of two or more independent experiments run on different dates.

We saw an increase in the amount of IFUs/mL in stressed mice, and because of this, we wanted to examine how the immune system is responding to infection during stress conditions. This experiment was to determine the gene expression levels of β-AR subtypes in splenic CD4+ T cells of stressed and non-stressed mice during *C*. *muridarum* genital infection. We hypothesized that differential gene expression of β-AR subsets might play an essential role in modulating the immune function against chlamydia genital infection, which is altered during stressful conditions. To test this, we used real-time quantitative PCR based on fluorescence detection and threshold cycle (Ct) values comparison to measure the mRNA level of β-AR subsets. As shown in the amplification curve (**Fig 2A**), Ct values of β2-AR in splenic CD4+T cells of stressed mice were approximately five times lower than that of non-stressed mice. In contrast, the amplification curve of β1-AR and β3-AR of splenic CD4+T cells showed no difference between stressed and non-stressed mice (**Fig 2B and 2C**). The relative expression of β2-AR in splenic CD4+T cells compared to that of non-stressed mice showed the highest fold-changes (p <0.05), which suggests that stress specifically up-regulates β2-AR expression on CD4+ T cells.

### Cold-induced stress leads to increased gene expression of GATA-3 in CD4+ T cells of the genital tract during *C*. *muridarum* genital infection

**Fig 3:**
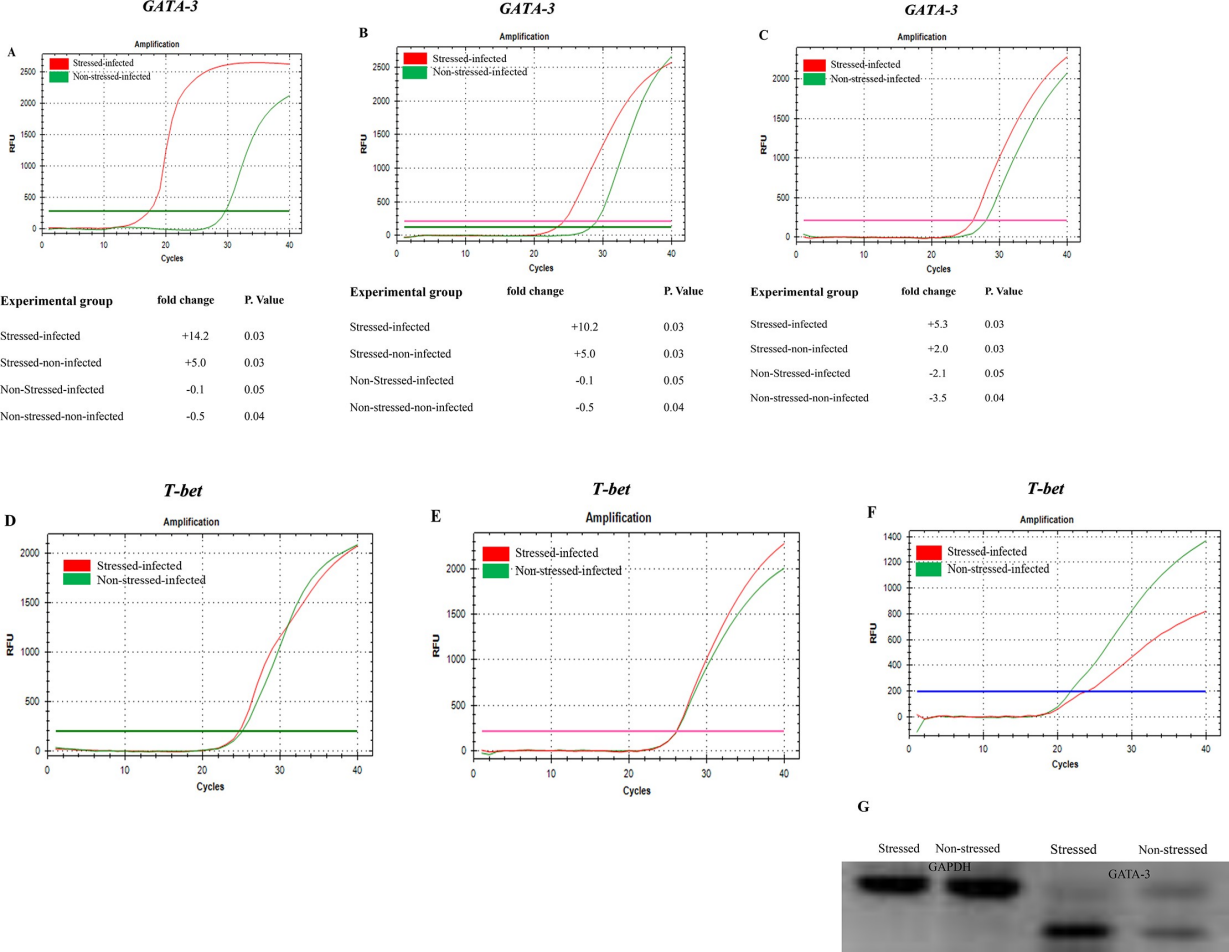
Gene expression profiles of GATA-3 and T-bet in T cells isolated from the whole or the parts of the genital tract of stressed and non-stressed mice during *Chlamydia muridarum* genital infection. Amplification cures and fold-changes of mRNA levels of transcription factors of T cells isolated **A**) whole genital tract, **B**) uterus horns, **C**) oviduct, **D**) T-bet in T cells of the whole genital tract, **E**) uterus horns **F** oviduct), and **G**) gel electrophoresis of GATA-3 are shown. ***Abbs***: **St** = stressed, **Non-St** = none-stressed. Shown data are representatives of two or more independent experiments.

Previous studies have shown that transcription factors T-box expressed in T cells (T-bet) and GATA-binding protein-3 (GATA-3) play essential regulatory roles in the differentiation of naive Th cells towards Th1 or Th2 cells in human subjects and animal models [18,51,52]. Modulation of cytokines and transcription factors (T-bet and GATA-3) in CD4 enriched cervical cells of *Chlamydia trachomatis* infected fertile and infertile women upon stimulation with chlamydial inclusion membrane proteins B and C was reported [17]. However, their expression patterns in CD4+ T cells in our stress model during chlamydia genital infection yet is unknown. This study was designed to determine the gene expression profiles of T-bet and GATA-3 in all populations of CD4+ T cells isolated from the spleen and genital tract of stressed and non-stressed mice with or without *C*. *muridarum* genital infection. We hypothesized that CIS alters the gene expression patterns of T-bet and GATA-3 that determine the production of Th1 and Th2 type cytokines. As shown in **Fig 3A**, a marked increase in gene expression of GATA-3 was obtained in CD4+T cells isolated from the genital tract of stressed mice compared to that of non-stressed mice. Furthermore, a significant increase in gene expression of GATA-3 in CD4+ T cells isolated from the uterus (**Fig 3B**) and cervix (**Fig 3C**) of stressed mice is shown compared to that of non-stressed mice. In contrast, T-bet mRNA expression was not elevated in either the whole genital tract or specific regions of the genital tract of either stressed or non-stressed mice (**Fig 3D and 3E**). Furthermore, T-bet gene expression was down-regulated in oviduct CD4+ T cells of stressed mice compared to that of non-stressed mice (**Fig 3F**). DNA intensity of the GATA-3 PCR end-product was significantly higher in stressed mice than non-stressed mice (**Fig 3G**). These results suggest that the switching of GATA-3 may lead to the switching of Th1 to Th2 immune system during stressful conditions of chlamydia genital infection.

### Cold-induced stress leads to increased gene expression of GATA-3 in the lysate of whole and regions of the genital tract of stress mice during *C*. *muridarum* infection

We observed a significant increase in gene expression of GATA-3 and a decrease in T-bet in CD4+ T cells of stressed mice. Then we want to investigate whether there is a differential gene expression of these Th1 and Th2 transcription factors in lysates of the different regions of the genital tract during *C*. *muridarum* genital infection. Our results show that after 48 hours of infection, CIS results in an up-regulated expression of GATA-3 in the oviduct, uterine horns, and cervix lysates (**Table 1**). In contrast, CIS resulted in the down-regulated gene expression of T-bet in the uterus and uterine horns of stressed mice compared to that of non-stressed mice (**Table 2**). Gene expression of T-bet in the cervix was undetectable. Our results indicate a positive correlation in gene expression patterns of transcription factors in CD4+ T cells and genital tract lysates of stressed and non-stressed mice.

**Table 1:** Fold-changes of gene expression of GATA-3 in lysates of the whole or parts of the genital tract of stressed or non-stressed mice during *C*. *muridarum* genital infection. Data shown are representative of two or more independent experiments performed on different dates.

Experimental group	fold change^a^	P. Value^b^	Isolated CD4+ T cells^c^
Stressed-infected	+6.2	0.03	**Oviduct**
Stressed-non-infected	+2.0	0.03	
Non-Stressed-infected	-2.1	0.05	
Non-stressed-non-infected	-4.5	0.04	
Stressed-infected	+10.2	0.03	**Uterus horns**
Stressed-non-infected	+3.0	0.03	
Non-Stressed-infected	-0.2	0.05	
Non-stressed-non-infected	-0.3	0.04	
Stressed-infected	+10.2	0.03	**Cervix**
Stressed-non-infected	+7.0	0.03	
Non-Stressed-infected	-2.0	0.05	
Non-stressed-non-infected	-3.5	0.04	

^a^ Fold change values of T-bet are a representative of two independent experiments normalized with GAPDH. We considered positive fold change values to indicate upregulation, whereas negative values indicate downregulation as compared to non-stressed mice.

^b^ p-value <0.05 shows a significant statistical difference compared to the control.

^c^ Antibody-negative selection method was used to collect CD4+ T cells from the different regions of the mouse genital tract.

**Table 2:** Fold-changes of gene expression of T-bet in lysates of the whole or parts of the genital tract of stressed or non-stressed mice during *C*. *muridarum* genital infection. T-bet expression in the oviduct and uterus horns is shown, but gene expression of T-bet in the cervix of stressed or non-stressed mice was undetectable.

Experimental group	fold change^a^	P. Value^b^	Isolated CD4+ T cells^c^
Stressed-infected	+1.0	0.04	
Stressed-non-infected	+1.0	0.03	**Oviduct**
Non-Stressed-infected	+8.5	0.03	
Non-stressed-non-infected	+6.0	0.03	
Stressed-infected	+3.2	0.05	
Stressed-non-infected	+1.0	0.03	**Uterus horns**
Non-Stressed-infected	+7.2	0.04	
Non-stressed-non-infected	+4.7	0.05	

^a^ Fold change values of T-bet are a representative of two independent experiments normalized with GAPDH. We considered positive fold change values to indicate upregulation, whereas negative values indicate downregulation as compared to control.

^b^ p-value <0.05 shows a significant statistical difference compared to the control.

^c^ Antibody-negative selection method was used to collect CD4+ T cells from the different regions of the mouse genital tract.

### Cold-induced stress alters gene expression patterns of transcription factors and signature cytokines in murine splenic naïve CD4+ T cells during *C*. *muridarum* genital infection

**Fig 4:**
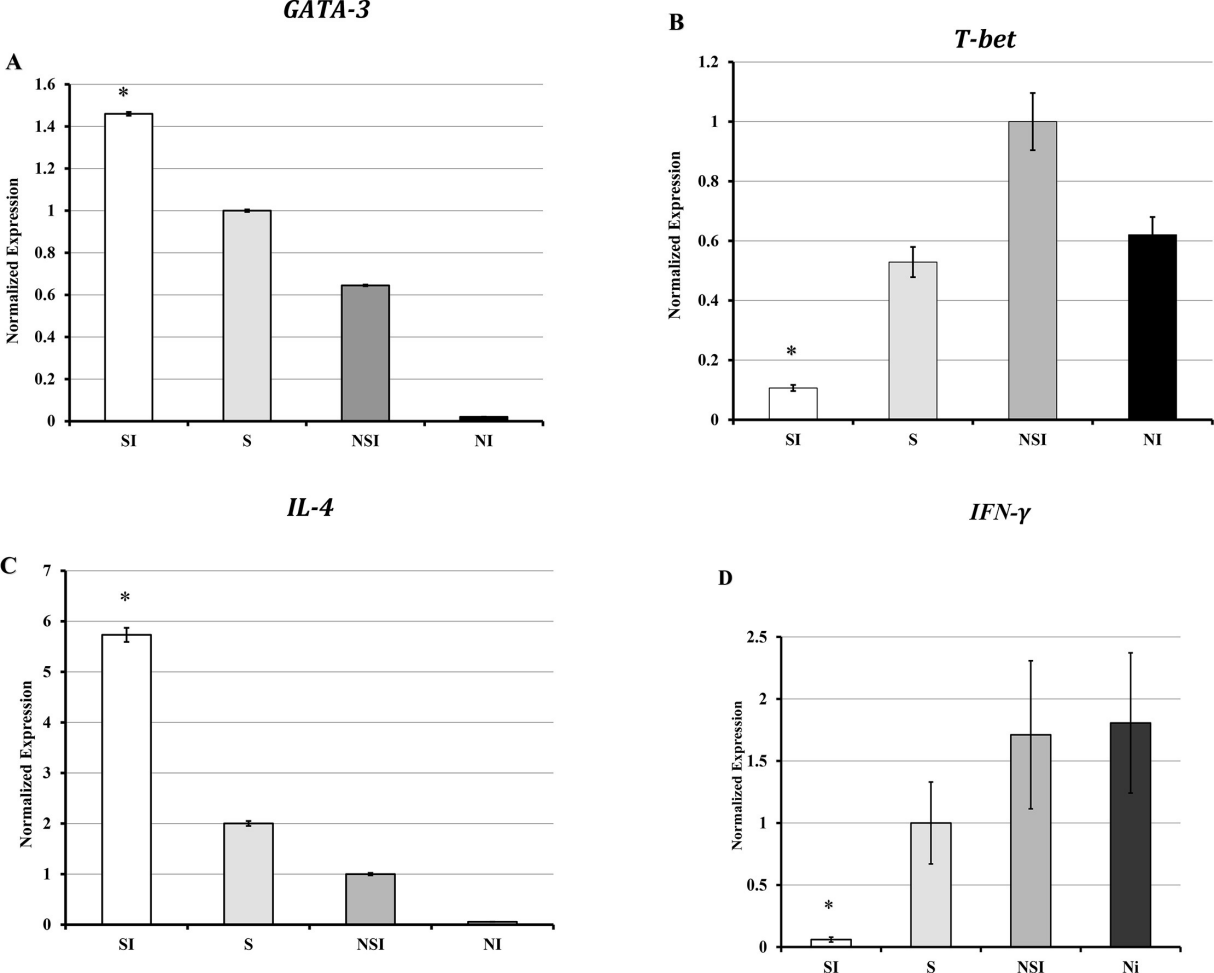
Fold-changes of gene expression of transcription factors and signature cytokines of Th1 or Th2. **A**) GATA-3, **B**) T-bet, **C**) IL-4) and **D**) IFN-γ in naïve CD4+ T cells isolated from spleen cells normalized to the internal control gene, GAPDH compared to the non-stressed mice are shown. Data shown a mean +/_ SEM of two or more independent experiments ran on different dates. *Denotes significant statistical differences between treatment groups at the level of (p < 0.05).

T-bet and GATA-3 are known to regulate the fate of naïve CD4+ T cells to produce Th1 or Th2 cytokines (IFN-γ, or IL-4, respectively [52]. We aimed to evaluate the gene expression of GATA3, T-bet, IL-4, and IFN-γ in splenic naïve CD4+ T cells isolated from stressed and non-stressed mice with or without *C*. *muridarum* genital infection. As shown in **Fig 4A** and **4C**, the normalized mRNA levels of GATA-3 and IL-4 in CD+4 T cells displayed significantly increased expression in stressed and infected mice. In contrast, T-bet and IFN-γ in CD+4 T cells displayed decreased expression considerably in stressed and infected mice (**Fig 4B** and **4D**). Our results show a direct correlation between the Ct values and fold-changes of the transcription factors and the signature cytokines of Th1 and Th2 cells. Overexpression of patterns of GATA-3 and IL-4 in stressed mice or the reduced expression of T-bet and IFN-γ in non-stressed mice was observed.

### Persistence of stress effect on the modulation of gene expression of transcription factors and secretion of Cytokines

**Fig 5:**
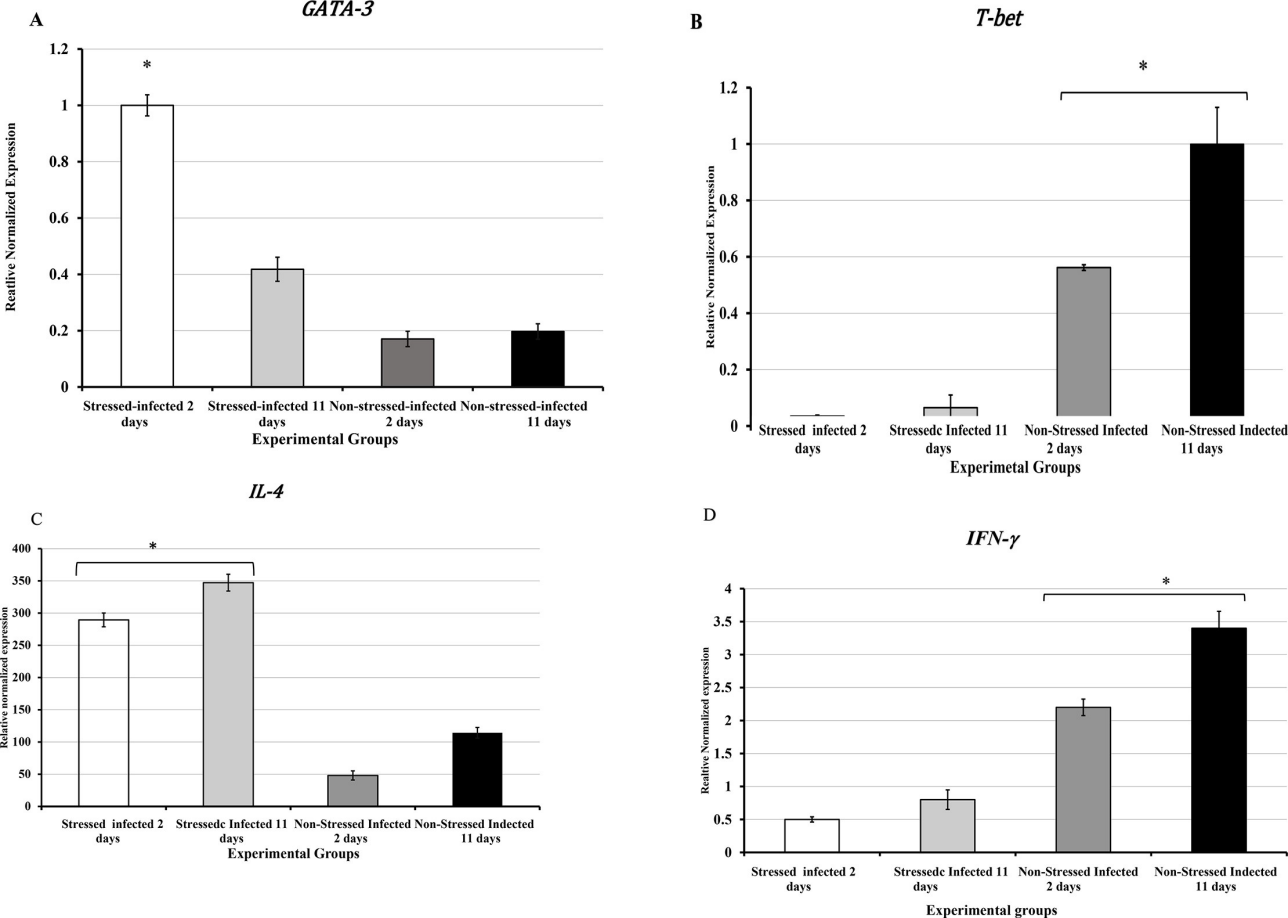
The persistence of the effect of CIS on the modulation of transcription factors and signature cytokines of Th1 and Th2 cells in the genital tract of stressed/non-stressed mice. Gene expression of **A**), GATA-3, **B**) T-bet, IL-4 **C**), and **D**) IFN-γ during two or 11-days post-*C*. *muridarum* genital infection relative to non-infected mice. Results are mean +/- SEM of two independently performed experiments. *Denotes significant statistical differences between treatment groups at the level of (p < 0.05).

It is well known that prolonged exposure to stress is a risk factor that subsequently leads to infection [36,38]. To determine how long the effect of stress persists, we compared the gene expression of T-bet and GATA-3 or secretion of IFN-γ and IL-4 by CD4+ T cells at two- or 11-days post-infection. As shown in **Fig 5A**, a marked increase in the expression of GATA-3 was demonstrated on day two. The gene expression of GATA-3 was significantly decreased at day 11 in SI mice but higher compared to that of NSI mice (p<0.05). As shown in **Fig 5B**, a marked decrease in expression of mRNA encoding T-bet remained constant at day two through day 11 in SI mice compared to NSI mice (p< 0.05). In contrast, normalized gene expression of IL-4 on day two was high and further increased at day 11 compared to the NSI group (**Fig 5C**). Similarly, the T-bet gene expression profile of IFN-γ remained significantly low at days two through 11 in SI mice compared to the NI mice (**Fig 5D**). Our findings indicate that the protracted chronic stress and *C*. *muridarum* genital infection induces the up-regulation of GATA-3 and IL-4, perhaps leading to the induction of Th2 and suppression of Th1 CD4+ T cells.

### Pre-exposure of splenic murine naïve CD4+ T cells to β2-AR agonist or antagonist alters gene expression patterns of transcription factors and T1 and Thr2 signature cytokines

Bone marrow-derived dendritic cells and CD4+ T cells express the β2-AR, which is the receptor of NE (53–55). The purpose of this experiment decided to examine the influence of adrenergic receptor agonist and antagonist to provide evidence of the involvement of β2-AR in Th and BMDCs differentiation. The action of Fenoterol and ICI118,551 as an agonist and antagonist respectively on the gene expression of T-bet & GATA-3 and IFN-γ and IL-4 of splenic naïve CD4+ T cells were tested. As shown in **Table 3**, The treatment of naïve CD4+ T cells with Fenoterol resulted in a marked increase of IL-4 gene expression compared to untreated CD4+ T cells. In contrast, exposure of naïve CD4+ T cells to ICI118, 551, resulted in elevated IL-12 gene expression. On the other hand, the treatment of naïve CD4+ T cells with Fenoterol resulted in a marked decrease in T-bet gene expression compared to control cells. In contrast, exposure of naïve CD4+ T cells to ICI118, 551, resulted in restoring gene expression of T-bet in stressed mice. Similarly, in the presence of Fenoterol, the fold change value of IL-4 of stressed mice was high, which is directly correlated with up-regulated gene expression compared to all the treatment groups.

**Table 3:** Impact of b2—AR agonist or b2-AR antagonist on gene expression of signature cytokines of Th1 and Th2 cells during *C*. *muridarum* genital infection of a murine stress model.

Chemical used	fold change^a^	P. Value^b^	Transcription factor^c^
Fenoterol	+2.2	0.04	
ICI118,551	+0.1	0.02	**GATA-3**
Concavalin-A	+1.0	0.03	
Cells only	0	0.05	
Fenoterol	+0.2	0.04	
ICI118,551	+1.0	0.02	**T-bet**
Concavalin-A	+1.1	0.03	
Cells only	0	0.03	
Fenoterol	+7.1	0.04	
ICI118,551	+1.1	0.02	**IL-4**
Concavalin-A	+1.0	0.03	
Cells only	0	0.05	
Fenoterol	-4.2	0.04	
ICI118,551	+1.1	0.02	**IFN-γ**
Concavalin-A	+1.0	0.03	
Cells only	0	0.05	

^a^ Fold change values of transcription factors and signature cytokines of Th1 and Th2 T cells are the mean of two independent qPCR experiments normalized with GAPDH. We considered positive fold change values to indicate upregulation, whereas negative values indicate downregulation as compared to control.

^b^ p-value <0.05 shows a significant statistical difference compared to the control.

^c^ Antibody-negative selection was used to purify CD4+ T cells from the different regions of the mouse genital tract.

### Pre-exposure of naïve splenic CD4+ T cells to β2-Ar agonist or β2-antagonist leads to differential production of Th1 and Th2 signature cytokines

**Fig 6:**
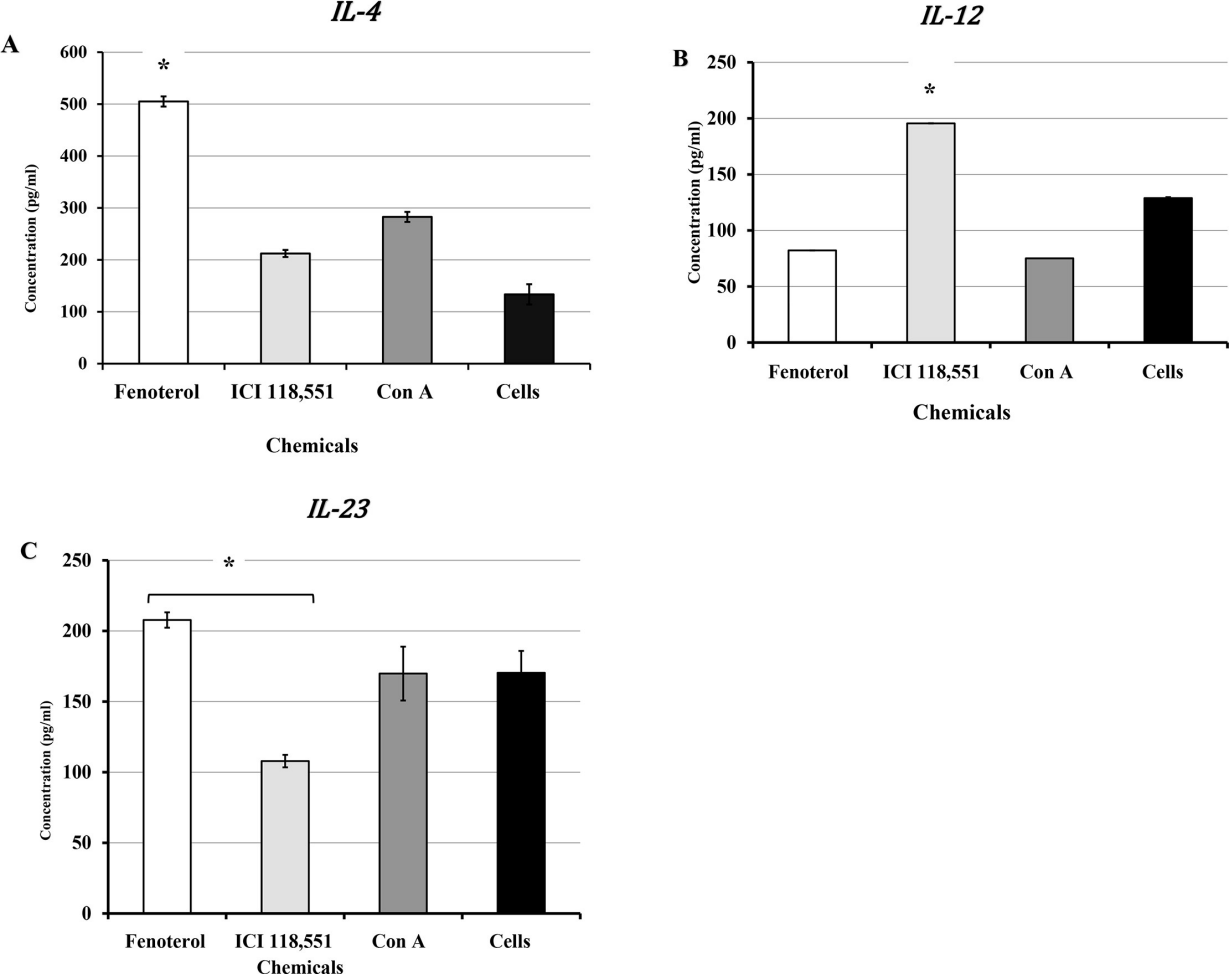
Presence of β2-AR agonist or antagonist on *in vitro* production of **A**) IL-4, **B**) IL-12, and **C**) IL-23 in naïve CD4 T-cells isolated from stressed non-infected mice. Immature dendritic cell culture was pre-exposed to NE (1 μM), Fenoterol (1 μM) and ICI 118,551 (1 μM) for 1 h before stimulation with LPS (5 μg/mL) for 24 h before RNA isolation. Data shown are a mean +/_ SEM of two or more independent experiments performed on different dates. *Denotes significant statistical differences between treatment groups at the level of (p < 0.05).

The production of IL-4 and IFN-γ after exposure of CD4+ T cells to Fenoterol or ICI118,551was evaluated. As shown in **Fig 6A**, IL-4 production in naïve CD4+ T cells was substantially increased with exposure to Fenoterol. In contrast, pre-exposure of CD4+ T cells to ICI118,551 resulted in a significantly reduced production of IL-4 (p<0.05). As shown in **Fig 6B**, a decreased production of IL-12 in naïve CD4+ T cells following pre-exposure ured to Fenoterol, whereas pre-exposure to ICI118,551 resulted in a significantly increased production of IL-12 (p <0.05). Production of IL-23 in naïve CD4+ T cells was substantially increased in the presence of Fenoterol but decreased in the presence of ICI118,551(**Fig 6C**). This data suggests that β2-AR signaling may play an essential role in modulating CD4+ Tells function as a critical regulator of the immune system during chlamydia genital infection.

### Polarization of T1 and Th2 cytokine production in a CIS murine model during *C*. *muridarum* genital infection

**Fig 7:**
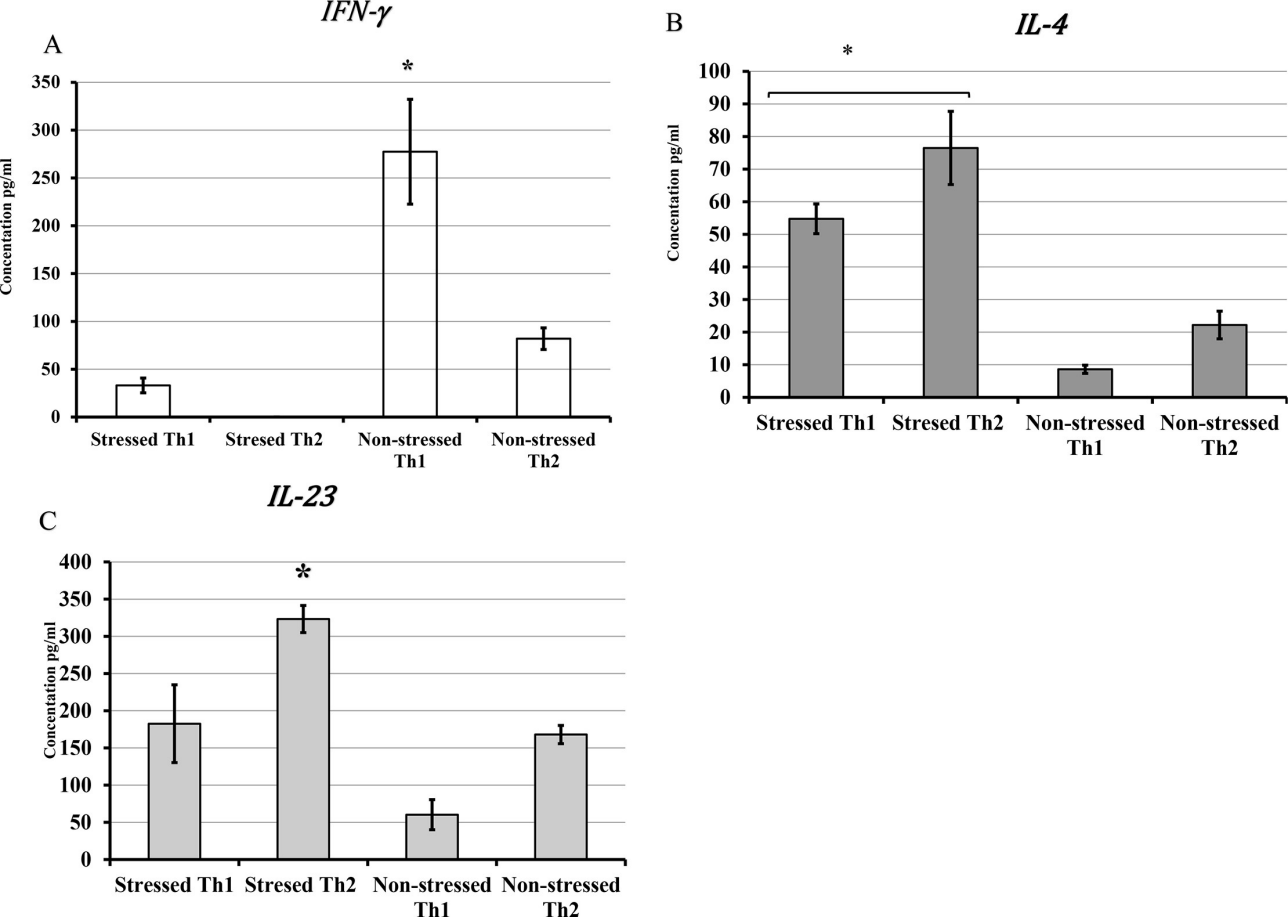
Polarization of naïve CD4+ cells in the production of signature cytokines of Th1 and Th2 cells determined by ELISA. **A)** IFN-γ, **B)** IL-4, **C**) IL-23. For differentiation of Th1 cells, murine IL-12, murine IFN-γ, human IL-2, and murine anti-IL-4 were added to cell cultures in plates precoated with murine anti-CD3 for stimulation. For Th2 cells, murine IL-4, murine anti-IFN-γ and murine and anti-IL-12 were added in plates precoated with murine anti-CD3 for stimulation. The production of cytokines was determined by ELISA. Data shown is a mean +/_ SEM of two or more independent experiments performed on different dates.

Because of the dichotomy of Th1 and Th2 cells remain as a significant area to investigate in our model, polarizing cytokines were used for Th subset differentiation *in vitro* (56,57). The purpose of this study was to evaluate the effect of cold-stress in altering the dynamics of Th1 and Th2 cytokine during *C*. *muridarum* genital infection. We hypothesized that cold stress alters the differentiation and variation in the switching of cytokine productions in CD4+T cells. T cells isolated from the genital tract were cultured in the presence of either Th1 cytokines and anti-IL-4 antibodies or Th2 cytokines and anti-IFN-γ antibodies added to cell cultures in plates precoated with murine anti-CD3 antibodies. As shown in **Fig 7A**, no polarization of IFN-γ was obtained with the addition of external Th1 cytokines and ant-IL-4 antibodies. However, increased IL-4 production was achieved with the addition of IL-4, anti-murine IFN-γ (10 ng/mL), and anti-murine IL-12 (**Fig 7B**). As shown in **Fig 7C**, the polarization of IL-23 was high with the addition of external IL-4 antibodies and anti-murine IFN-γ (10 ng/mL), and anti-murine IL-12. These results indicate that CIS impairs the stimulation that drives polarization of T cells toward IFN-γ production but not IL-4 or IL-23 production.

### Western blot analysis on the secretion of β2-AR, transcription factors and signal transducer and activator of transcriptions (STATs)

**Fig 8:**
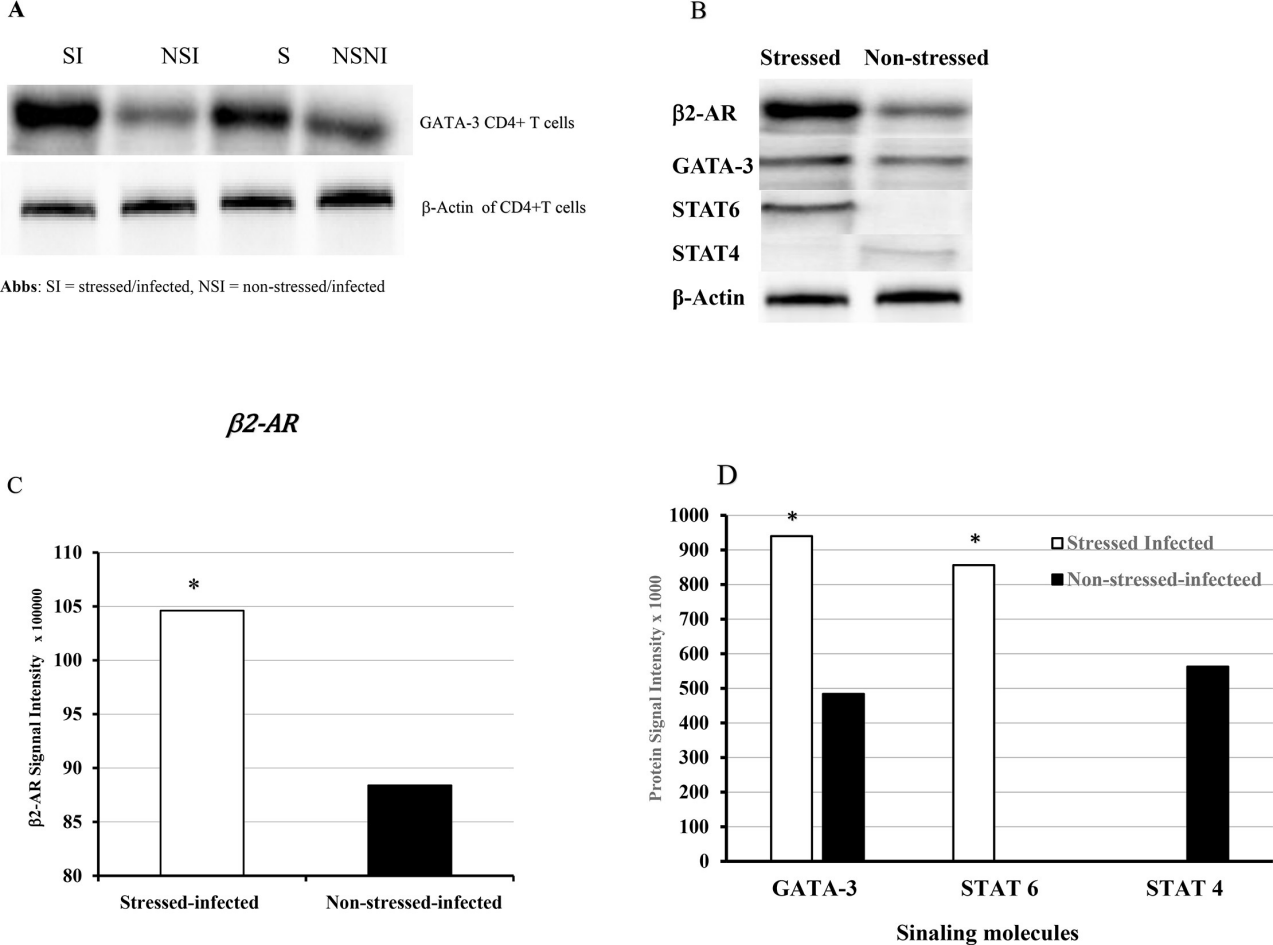
Western blot analysis of Protein expression of **A**) GATA-3, **B**) β2-AR, GATA-3, STAT 4 & 6, and β-Actin, **C**) β2-AR intensity, **D**) GATA-3, STAT4 and STAT6 intensities in CD4+T cells isolated from the genital tract of stressed and non-stressed mice. Amplified Opti-4CN Substrate Kit from Bio-Rad was used to detect protein concentration following the manufacturer’s instructions. Relative protein expression was calculated as described in materials and methods. Data shown are a representative of two or more independent experiments performed on different dates.

We continued to investigate the differential expression of T-bet and GATA-3 in CD4+ T cells of stressed and non-stressed mice using Western blot analysis. Besides, changes in expression of β2-AR signal transducer and activator of transcription (STAT) 4, and 6 were examined by Western bot analysis. As shown in **Fig 8**, exposure to CIS resulted in increased and reduced secretion of GATA-3 and T-bet, respectively, in the genital tract of stressed mice compared to that of non-stressed mice. Furthermore, high intensity of STAT 6 was observed in CD4+ cells of stressed mice compared to low intensity of STAT 4 of T-bet in CD4+ T cells of non-stressed mice. The results suggest that GATA3 and STAT6 are essential for switching of Th1 to the Th2 subtypes. CD4+ development skewed toward Th2 differentiation, with subsequent down-regulation of STAT4, T-bet, levels in CD4+ T cells during a CIS situation.

### Cold-induced stress results in differential gene expression of β-AR subtypes in splenic dendritic cells

Previous studies have shown that DCs have an essential role in the induction of T cell responses to clear *C*. *muridarum* during genital infection [29,53]. However, the influence of CIS on DCs function is not defined. Because β-ARs have differential patterns of expression on immune cells, we continued investigating the gene expression patterns of β-AR subtypes in BMDCs of our murine stress model. We hypothesized that cold induced-stress results in increased gene expression of β2-AR and decreased gene expression of β1-AR and β3-ARs in BMDCs. As shown in **Table 4**, fold changes in gene expression of β2-AR in LPS-stimulated in BMDCs was slightly up-regulated in stressed mice compared to the control. On the other hand, stress and stimulation with LPS resulted in little or no difference in gene expression of β1-AR and β3-AR in BMDCs.

**Table 4:** Gene expression profiles of β-AR subtypes in BMDCs of stressed and non-stressed mice.

Experimental group	fold change^a^	P. Value^b^	Beta-adrenergic receptor (β-AR) subsets
Stressed + LPS	+2.0	0.05	
Stressed only	+1.5	0.02	**β2-AR**
Non-Stressed + LPS	+1.9	0.02	
Non-stressed cells only	+1.0	0.05	
Stressed + LPS	-0.86	0.01	
Stressed only	-0.9	0.05	**β1-AR**
Non-Stressed + LPS	+1.0	0.05	
Non-stressed cells only	+0.76	0.05	
Stressed + LPS	-1.50	0.03	
Stressed only	-1.30	0.05	**β2-AR**
Non-Stressed + LPS	+1.3	0.01	
Non-stressed cells only	-1.0	0.05	

^a^ Fold-change values of b-AR subtypes are a representative of two or more independent qPCR.

experiments normalized with GAPDH. The antibody-negative selection method was used to purify matured bone marrow-derived dendritic cells. We considered positive fold change values to indicate upregulation, whereas negative values indicate downregulation as compared to non-stressed mice.

^b^ P-value <0.05 shows a significant statistical difference compared to the control.

### Norepinephrine, β2-AR agonist, or β2-AR antagonist modulates the differentiation of BMDCs cultured *in vitro*

**Fig 9:**
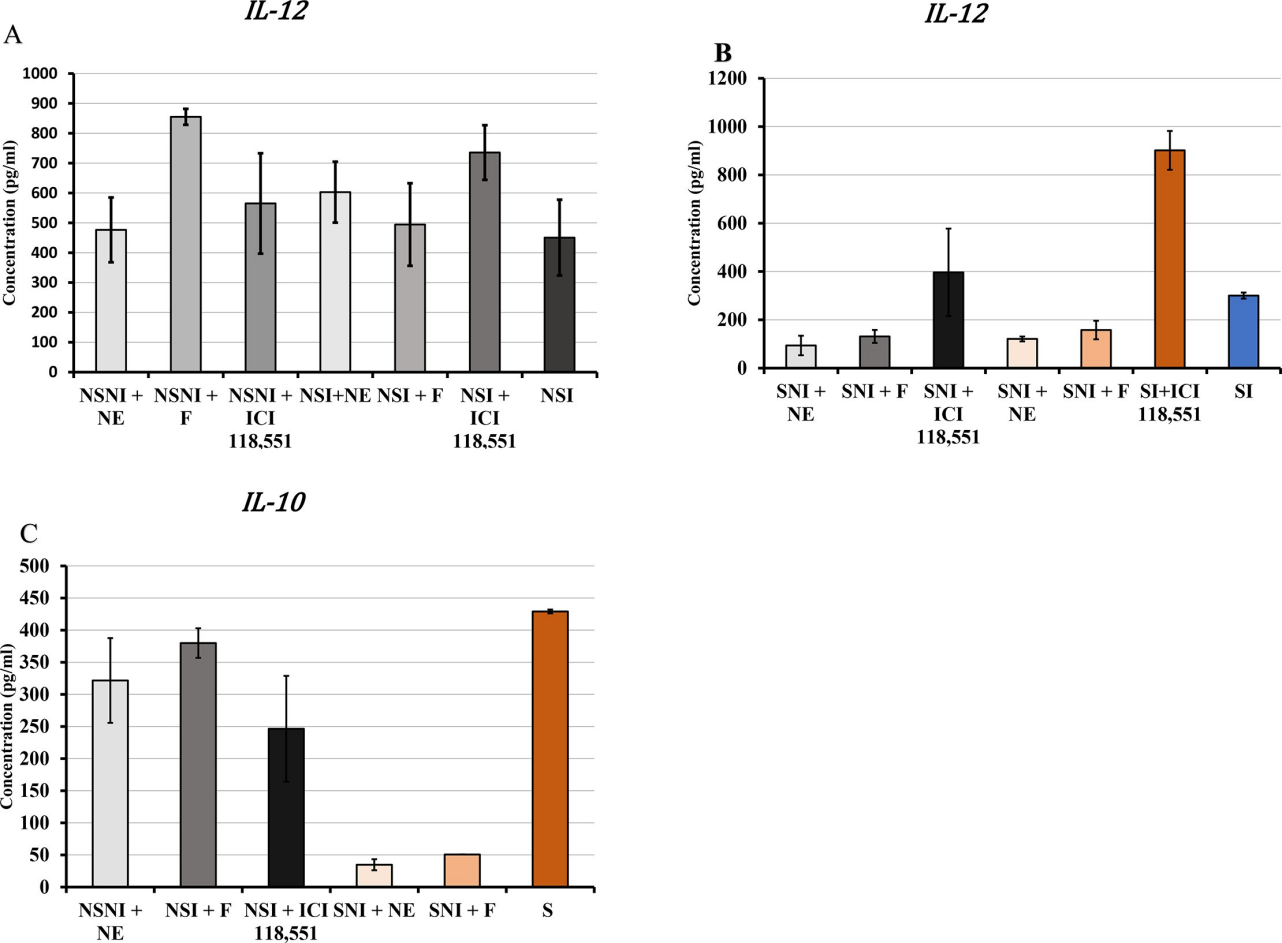
Differential cytokine production of immature BMDCs pre-exposed to NE, Fenoterol, or ICI118,551. **A**) non-stressed-non-infected (NSNI) or non-stressed-infected mice(NSI); **B, C**) Stressed-non-infected (SNI) or stressed-infected (SI) mice; to NE, Fenoterol (F), or ICI118,551 results in irregular production IL-12 and IL-10 production. Immature dendritic cell culture was pre-exposed to NE (1 μM), Fenoterol (1 μM) or ICI 118, (1 μM) for 1 h before stimulation with LPS (2.5 μg/mL) for 24 h before RNA isolation. Data shown are a mean +/_ SEM of two or more independent experiments performed on different dates. *Denotes significant statistical differences between treatment groups at the level of (p < 0.05).

Norepinephrine binds and stimulates β2-AR, which is predominantly expressed on CD4+ T cells and B cells [54,55]. Studies have further shown that production and direct binding of NE to β2-AR modulates DCs immune responses, including altering cytokine production, lymphocyte proliferation, and antibody secretion [56–58]. We are interested in examining the β2-AR stimulation of murine DCs on CD4+ T cell activation of stressed mice. Culturing of immature BMDCs in the presence or absence of NE, β2-AR agonist, and β2-AR antagonist *in vitro* before stimulation with LPS was tested. Pre-exposure of cells to Fenoterol resulted in a marked increase of IL-12 in NSNI mice compared to that of NSI mice (**Fig 9A**). However, ICI 118,551, treatment resulted in increased production of IL-12 by BMDCs of stressed mice compared to that of non-stressed mice. Pretreatment with NE or Fenoterol showed a significant decrease in IL-12 production between NSI and NSNI However, ICI 118,551 resulted in a significant increase in IL-12 production, particularly in stressed-infected mice (**Fig 9B**). Pretreatment of BMDCs obtained from stressed mice with NE, Fenoterol, or ICI118,551 resulted in significantly decreased production of IL-10 compared to that of BMDCs obtained from NSNI mice (**Fig 9C**).

### Naïve T cells co-cultured with BMDCs pretreated with β2-AR agonists, and antagonists resulted in the production of different levels of Th1 and Th2 cytokine production

**Fig 10:**
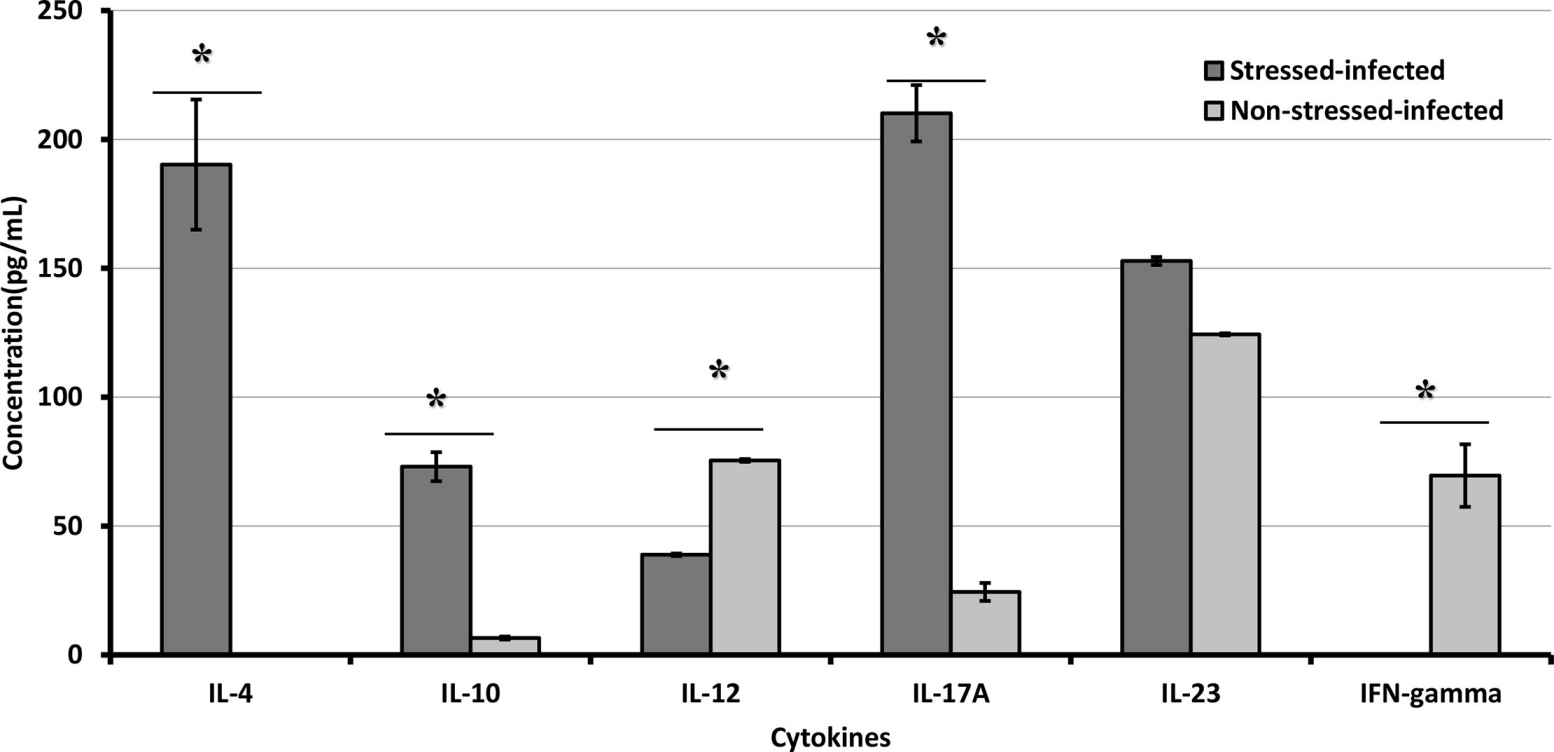
Cytokine production kinetics of CD4+T cells and dendritic cells co-cultured *in vitro*. CD4+ T cells from stressed and non-stressed mice isolated by negative selection were co-cultured for 48 h. Shown data are mean+/_SEM two or more independent experiments performed on separate dates. *Denotes statistically significant differences between stressed and non-stress mice.

It is known that DCs have an essential role in the induction of T cell responses to clear *C*. *muridarum* during genital infection [29,53]. To clarify how CIS affects the function of DCs in our murine model, we determined the ability of BMDCs-CD4+ T cell co-culturing to produce cytokines. Then, our results show that BMDCs pre-treated with NE or Fenoterol showed decreased IL-12 production compared to that of non-treated CD4+ T cells (**Fig 10**). Furthermore, the co-culturing of mature BMDCs and naïve CD4+ T cells resulted in increased production of IL-4, IL-10, IL-17, and IL-23 in culture supernatants, suggesting that stimulation of β2-AR receptor leads to increased production of Th2 cytokines (**Fig 10**). Our results demonstrate that CIS influences the activation and differentiation of BMDCs following stimulation by LPS, which might induce shifts in the Th1/Th2 ratio and thus impairing the production of protective Th1 cytokines.

### Stimulation of a mixed population of Th1 and Th2 CD4+ T cells using anti-CD3 antibodies in the presence of β2-AR agonist or β2-AR antagonists yielded varying observations

The new approach to stimulate CD4+ T cells proliferation *in vitro* is through coating plates with anti-CD3 has become a standard method [42,59,60]. The effect of CIS on antigen-dependent or -independent stimulation of CD4+ T cells based on the binding of anti-CD3 antibodies to CD3 activating TCR complex is not defined. The impact of β2-AR agonist or β2-AR antagonists on CD4+ T cell activation through the stimulation with ant-CD3 antibodies was examined (**Table 5**). The β2-AR agonist resulted in a complete blockage of IL-12 production and a significant blockage of IL-4 production, regardless of stress treatment and plate coating with the anti-CD3 coating (p<0.5). β2-AR agonist, Fenoterol, and unexpectedly the β2-AR antagonist, ICI118, 551, resulted in a substantial increase of IL-4 production in stressed mice but not in plates without anti-CD3 coating (p<0.5). The production of IL-10 in stressed mice was the maximum in plates coated with antiCD3 (p<0.5). Production of IL-23 was high regardless of stress/no stress conditions and plate coating with ani-CD3 antibodies. It is well known that ant-CD3 antibodies could be both immunosuppressive and immunostimulant to CD4+ T cells. Thus, further study of the activating effects of anti-CD3 antibodies can probably enhance the understanding of the mechanism of in vitro CD4+ T cell activation.

**Table 5:** Effect of β2-AR agonist or β2-AR antagonist on the production of Th1 and Th2 cytokines by CD4+ T cells of stressed/non-stressed mice co-cultured with BMDCs on plates with/without anti-CD3 antibodies. Data are representative of two or more independent experiments performed on different dates.

BMDC-CD4+ T cell Co-culturing	**None anti-CD3 antibody-coated plates**							
	**Stressed**				**Non-stressed**			
	IL-4	IL-10	IL-12	IL-23	IL-4	IL-10	IL-12	IL-23
ICI 188,551+^**a**^	37	123	**ND**^**c**^	191	**ND**	53	**ND**	126
Fenoterol+^**b**^	192	104	74	160	107	54	141	118
CD4+ T cells only	64	70	**ND**	88	**ND**	62	128	110
BMDC-CD4+ T cell co-culturing	**Anti-CD3 antibodies coated plates**							
	**Stressed**				**Non-stressed**			
	IL-4	IL-10	IL-12	IL-23	IL-4	IL-10	IL-12	IL-23
ICI 188,551+^**a**^	26	74	**ND**	172	**ND**	49	**ND**	157
Fenoterol+^**b**^	36	577^d^	**ND**	248	44	60	48	140
CD4+ T cells only	38	101	**ND**	74	**ND**	64	75	98

^a^ICI 188,551, resulted in a complete blockage of IL-12 production, whereas a statistically IL-123 significant output.

^b^Fenoterol, resulted in a substantial increase of IL-4 production in stressed mice in plates without anti-CD3 antibodies (p<0.5).

^c^ND indicates a non-detectable level of cytokines by ELISA

^d^ One-way ANOVA was used to determine group differences.

### Interconnection of genes and their products that may be essential during stress and genital infection

**Fig 11:**
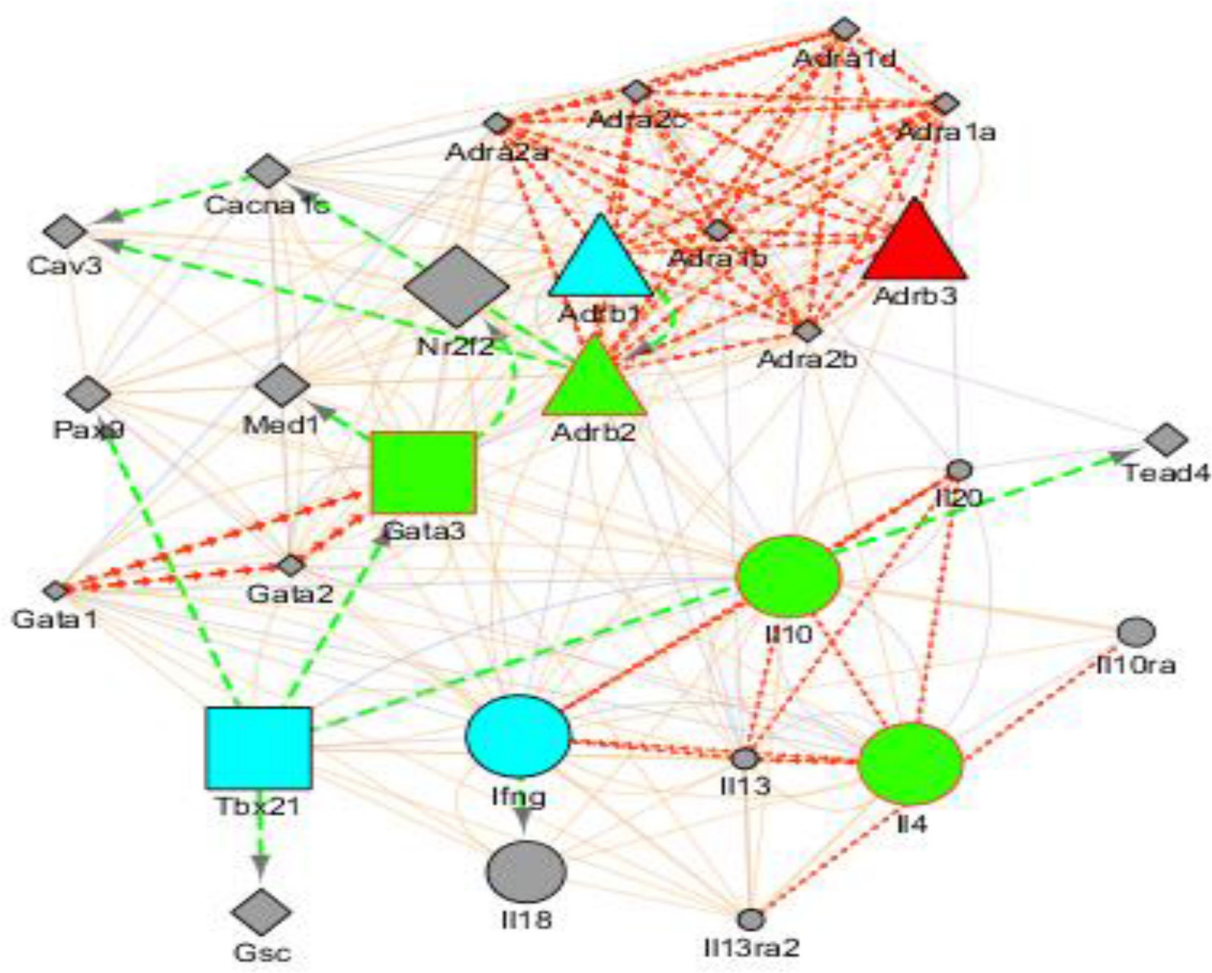
Cystoscope image of functional gene expression and potential regulation during *C*. *muridarum* genital infection in mice. The color of the nodes shows up- or down-regulation of genes in stressed mice during *C*. *muridarum* genital infection. Nodes shapes are made to show transcription factors, cell signaling receptors, and cytokines. The edges are colored to show nodes that are affected by up-and down-regulation in stressed mice during *C*. *muridarum* genital infection.

Up-regulated or down-regulated gene expression profiles from stressed and non-stressed mice during *C*. *muridarum* genital infection were used to generate an interaction network to identify any other gene that could also be affected by their expression. As shown in **Fig 11**, gene nodes that are colored blue were down-regulated and up-regulated during stress and non-stressed conditions, respectively. Gene nodes in green were up-regulated during stress and down-regulated during non-stress conditions. The gene node that is colored red was down-regulated during both in stressed and non-stressed mice. The node shape shows the type of function that the genes play during stressful conditions. Nodes that are square, circle, and triangles are transcription factors, cytokines, and cell signaling receptors, respectively. Line color and shape indicates the regulation that their expression could play on other genes during stressful conditions. Green dashed lines indicate the genes that could be down-regulated or up-regulated during stress or non-stressed conditions, respectively. Red arrowed lines show genes that could be up-regulated and down-regulated during stressed and non-stressed conditions, respectfully.

## Discussion

We previously showed that repeated exposure of mice to cold water leads to increased NE and EP production in plasma, spleen, and genital tract lysates that are speculated to increase the intensity of *C*. *muridarum* genital infection [49,50]. It is well known that T helper cells have critical protective roles during chlamydia genital infection (13–16); however, whether CIS contributes to the imbalance of Th1 and Th2 cells in our murine stress model is unknown. In the present study, we used the stress model to evaluate the differentiation of CD4+ T cells into Th1 and Th2 responses during *C*. *muridarum* genital infection. Gene expression profiles of β-AR subtypes on CD4+ T cells, T-bet, GATA-3, differentiation of BMDCs, and secretion of Th1 and Th2 cytokines were determined. Furthermore, stimulation of β2-AR by pre-exposure to its agonist and antagonist and *in vitro* polarization of naïve CD4+ T cells toward the Th1 or Th2 subset in the presence of Th1 or Th2 cytokines was examined.

Several previous studies indicate that the stress hormone, NE, suppresses the immune system through the stimulation of β2-AR expressed on various cell surfaces [39, 53,61]. In this study, we observed a dominant expression of β2-AR on CD4+ T cells isolated from the spleen and genital tract of stressed mice compared to that of non-stressed mice. Our finding is in line with numerous reports that have shown β2-AR is commonly expressed on the surface of immune cells, including Th1 cells, and when stimulated, it plays a significant role in the inhibition of IL-12 and IFN-γ production [42,61]. Our results further show that β2-AR expression is significantly higher in stressed and infected mice when compared to stressed mice only. However, the mechanisms that contribute to the stress-infection synergy, which results in increased gene expression of the β2-AR subtype, are unknown. These observations demonstrate that stress increases specifically the expression of β2-AR that is correlated significantly with increased C. *muridarum* infection, as evidenced by more significant shedding in stressed mice than the control mice [50]. This stress-infection model may provide a system to begin investigating the sequential relationship between stress or stress-infection combined synergy to identify mechanisms by which CIS alters susceptibility to *C*. *muridarum* genital infection. From our research, we speculate that CIS increases the expression of β2-AR in Th cells of stressed mice, which may play a significant role in the alteration of the immune response. In contrast, our findings suggest that CIS has little effect on the pattern of gene expression of β1-AR and β3-AR subtypes in splenic CD4+ T cells of the host.

Previous studies show NE suppresses the immune system through the stimulation of β2-AR expressed on various cell surfaces [39,62]. Because β2-AR is the most commonly expressed receptor in CD4+ T and DCs, we tested whether a β2-AR agonist or β2-AR antagonist affects cytokine production [55,62]. Our results show that the β2-AR agonist, Fenoterol, decreases Th1 cytokine production, probably by blocking T cell receptor (TCR)-mediated signaling, whereas the β2-AR antagonist. ICI118, 551, restores Th1 cytokine production. However, controversy exists on the effect of β2-AR agonist or β2-AR antagonists on Th2 cells, where studies have shown that β2-AR agonists modulate cell functions via direct actions on both Th1 and Th2 cells [30,59,62].

It is well documented that naïve CD4+ T cells are able to produce different patterns of cytokines throughout their stages of differentiation [53,56,64]. T-bet and GATA-3 are known to play critical roles in the differentiation of naive Th1 cells towards Th1 or Th2 cells in human subjects and animal models [60,62]. However, how CIS affects their differentiation into Th cell subsets is not well defined. Our results based on non-polarizing conditions of CD4+ T cell differentiation show that T-bet gene expression was low, whereas GATA-3 gene expression was markedly high in CD4+ T cells of stressed mice. These observations indicate that CIS results in a restricted pattern of gene expression of transcription factors of Th1 and Th2 cells. In contrast, other researchers under different conditions reported that effector/memory CD4+ T cells that can produce either Th1 or Th2 cytokines commonly co-express T-bet and GATA-3 [63,64].

Our study further revealed increased GATA-3 expression correlated with increased IL-4 expression/secretion in contrast to decreased expression of T-bet and IFN-γ expression/ secretion during *C*. *muridarum* genital infection. The increased expression of GATA-3 and IL-4 secretion of our study favors the dominance of Th2 cells, reflecting the switching of IFN-γ production by Th1 to IL-4 production by Th2 that probably subsequently result in the pathological development of chlamydia genital infection [13, 18]. These findings further confirm and extend previous reports from other research labs that stress treatment leads to up-regulated expression of GATA-3, whereas down-regulated expression of T-bet [17, 51, 52]. Moreover, significant secretion of IL-4 and STAT6 were shown in stressed mice compared to non-stressed mice. This positive correlation indicates that IL-4 probably stimulates STAT6 is acting as a transcription factor, activating genes responsible for the differentiation of Th2 cells. Thus, the IL-4-STAT-6 pathway is perhaps involved in activating and regulating Th2 immune response during CIS and chlamydia genital infection [39,60]. Our findings show prolonged CIS can persist in our murine stress model, having a lasting impact on suppression of the immune system even after the cessation of the stressing process. This observation also indicates that a long duration of stress is critical in promoting immune suppression in the implicated host [35,38]. This slow immune response recovery shown in our study is in line with several studies demonstrating that the CIS developed in our lab has long-lasting effects on the immune system that subsequently leads to infection [36, 38,40,47].

Dendritic cells are significantly crucial in antigen presentation activities to T cells for activation and differentiation of T cells to different subsets [54, 59]. However, the influence of CIS on differentiation and maturation of BMDCs and its activity in CD4+ T cell function is not well defined. For a better understanding of the effect of CIS, we tested the function of matured BMDCs on CD4+ T cell activation in combination by co-culturing *in vitro*. The co-culturing of DCs and CD4+ T cells showed decreased IL-12 and IFN-γ production but increased IL-10, IL-17, and IL-23 secretion. Our findings are in line with previous reports that have shown epinephrine-primed murine BMDCs facilitate the production of IL-17A and IL-4 but not IFN-γ by CD4+ T cells [65]. Furthermore, our findings are in line with previous studies that demonstrated β2-AR agonists modulate T cell functions via direct actions on Th1 and Th2 cells, where CD3 + CD28-stimulate IL-13 (Th2 cytokine) and IFN-γ and IL-2 (Th1 cytokines) production were inhibited by β2-AR agonist [54,59]. We feel that DCs play a crucial role in regulating CD4+ T cells function as we were able to demonstrate the dichotomy between IL-12 and IL-4 in the presence of β2-AR agonist and β2-AR antagonist. Our study on a mixed population of Th1 and Th2 resulted in the inhibition of Th1 cytokines but not Th2 cytokines; thus, our findings underscore the importance of further study on the action of β2-AR agonists and antagonists during stressful conditions.

In exploring more about the differentiation of CD4+ T cells during the CIS condition, the responsiveness of naïve CD4+ T cells to polarizing cytokines of Th1 and Th2 was determined as previously determined [66]. Polarization of Th1 and Th2 cells of stressed mice toward IFN-γ or IL-4 production using exogenous cytokine milieu resulted in no change of production of IFN-γ but slightly increased production of IL-4. This observation suggests that the conditions that trigger polarization of Th1 cells probably are not the only mechanism requiring IL-12 and STAT4 signaling. On the other hand, IL-4 was sufficient to derive Th2 differentiation, possibly through STAT 6 activation. Our findings implicate that stress effect selectively inhibits the differentiation of Th1 compared to that of Th2 development, but further study is warranted about the polarization of Th1 and Th2 cytokines. We feel that the use of Th1 and Th2 cells purified to approximately 100% homogeneity by FACS selection may provide the true polarization nature of Th1 and Th2 cytokines when stimulated with cytokines and antibodies.

It is speculated that anti-CD3 antibodies could be both immunosuppressive and stimulant to T cells [60]. The effect of CIS on whether antigen-dependent or -independent stimulation of CD4+ T cells is not known. For a better understanding of the activation of the TCR complex based on anti-CD3 antibody binding, stimulation of mixed populations of naive mouse CD4+T cells by culturing on anti-CD3 antibody-coated conditions was performed. The data slightly favored the production of Th2 cytokines without IL-12 detection even in the presence of β2-AR antagonist. These data suggest that stress-induced stress promotes the dominance of a Th2 dominated immune response during the culturing of mixed populations of naive Th1and Th2 cells. This observation suggests the optimization of *in vitro* expansion of CD4 T cells using anti-CD3 and co-stimulation with anti-CD28 is needed in future studies.

Nowadays, datasets are available in public archives such as the Gene Expression Omnibus (GEO) and cystoscope [67]. Although the scope of this study was not to undertake a detailed analysis of biological processes and signaling pathways of genes or their products, we inserted our findings into global datasets and constructed a network and identified the genes shown in **Fig 11**. We identified a group of proteins that have interconnection during chlamydia infection of the stressed model identified several candidate targets for further fundamental experimental research.

Literature shows the existence of functional differences in Th1 and Th2 responses as protective or suppressive, respectively, during chlamydia infection [15,17,18]. Still, studies focusing on the pathogenesis of chlamydia and host immune responses in animal models and human subjects are steadily increasing [30,68, 69]. However, the immune responses that promote chlamydia clearance or that cause immunological tissue damage remain to be explored. Although previous studies showed that β2-AR is absent in Th2 cells [55], we feel that CIS leads to Th2 dominated conditions that favor enhanced development of immunopathogenesis during *C*. *muridarum*. However, our findings are in line with other studies that demonstrated β2-AR agonists modulate T cell functions via direct actions on Th1 and Th2 cells, where CD3 + CD28-stimulate IL-13 (Th2 cytokine) and IFN-γ and IL-2 (Th1 cytokines) production were inhibited by β2-AR agonist [30,62]. Therefore, exploring the mechanisms that regulate β2-AR expression may lead to the development of methods for limiting Th1 or Th2 responsiveness to NE or β2-AR agonists that may lead to the development of CD4+T cells-mediated pathologies during chlamydia genital infection.

In summary, we undertook a detailed assessment of how CIS modulates a mixed population of CD4+ T and BMDC cells. A marked increase in expression of GATA-3, as determined by elevated section of IL-4, and decreased expression of T-bet, IL-12, and secretions of IFN-γ in CD4+ T cells of stressed mice compared to that of non-stressed mice were obtained. In contrast, increased production of IL-4, IL-10, IL-17, and IL-23, in culture supernatants of co-culture of CD4+ T cells and BMDCs were obtained, suggesting that stimulation of β2-AR receptor leads to the increased production of Th2 cytokines and decreased production of Th1 cytokines in stressed mice. The revealed changes in expression of β2-AR, transcription factors, signature cytokine production dynamics of Th1 and Th2 subsets may indicate CIS treatment leads to the imbalance between Th1 and Th2 type cells, which could be one of the major causes of the development of pathology and infertility associated with chlamydia genital infection [50]. Although there are multiple mechanisms that sympathetic nervous induced system of regulation [70], β2-AR signaling pathway may contribute to increased immunosuppression during stress chlamydia genital infection. We feel that the major stress response is NE production, which plays a key role in regulating CD4+ T cells and DCs function. The present finding, taken together within previous observations [49,50], indicates that cold-water induced stress increases the intensity of chlamydia genital infection in mice. The long-term goal of our study is to employ the cold-water induced stress in a β2-AR KO murine model, and the hypothesis to be tested is: cold water-induced stress leads to NE modulation of the immune response against *C*. *muridarum* genital infection by enhancing the production of immunopathogenic cytokines that result in disease sequelae.

## Supporting information

S1 Fig Gel electrophoresis profile of beta-1,-2,-3 adrenergic receptors (b-AR) gene expression in CD4+ T cells. (PDF)

S2 Fig Gel electrophoresis PCR products on gene expression of GATA-3 and T-bet from T cells isolated from genital tracks of *Chlamydia muridarum* infected mice. (PDF)

S3 Fig Western blot analysis of transcription factors in CD4 T cells isolated from stressed and non-stressed mice with/without *Chlamydia muridarum* genital infection. (PDF)
