# Correction: Modulation of T helper 1 and T helper 2 immune balance in a murine stress model during *Chlamydia muridarum* genital infection

**DOI:** 10.1371/journal.pone.0358082

**Published:** 2026-09-09

**Authors:** Tesfaye Belay, Elisha Martin, Gezelle Brown, Raenel Crenshaw, Julia Street, Ashleigh Freeman, Shane Musick, J Tyler Kinder

The eighth author’s name was incorrectly formatted. The correct name is: J Tyler Kinder.
