# Supplementary material for: Modulation of T helper 1 and T helper 2 immune balance in a murine stress model during Chlamydia muridarum genital infection
Source: PLoS One. 2020 May 15;15(5):e0226539. doi: 10.1371/journal.pone.0226539 (PMC7228091; doi:10.1371/journal.pone.0226539)
Supplement: S2 Fig — (PDF) [file pone.0226539.s002.pdf]

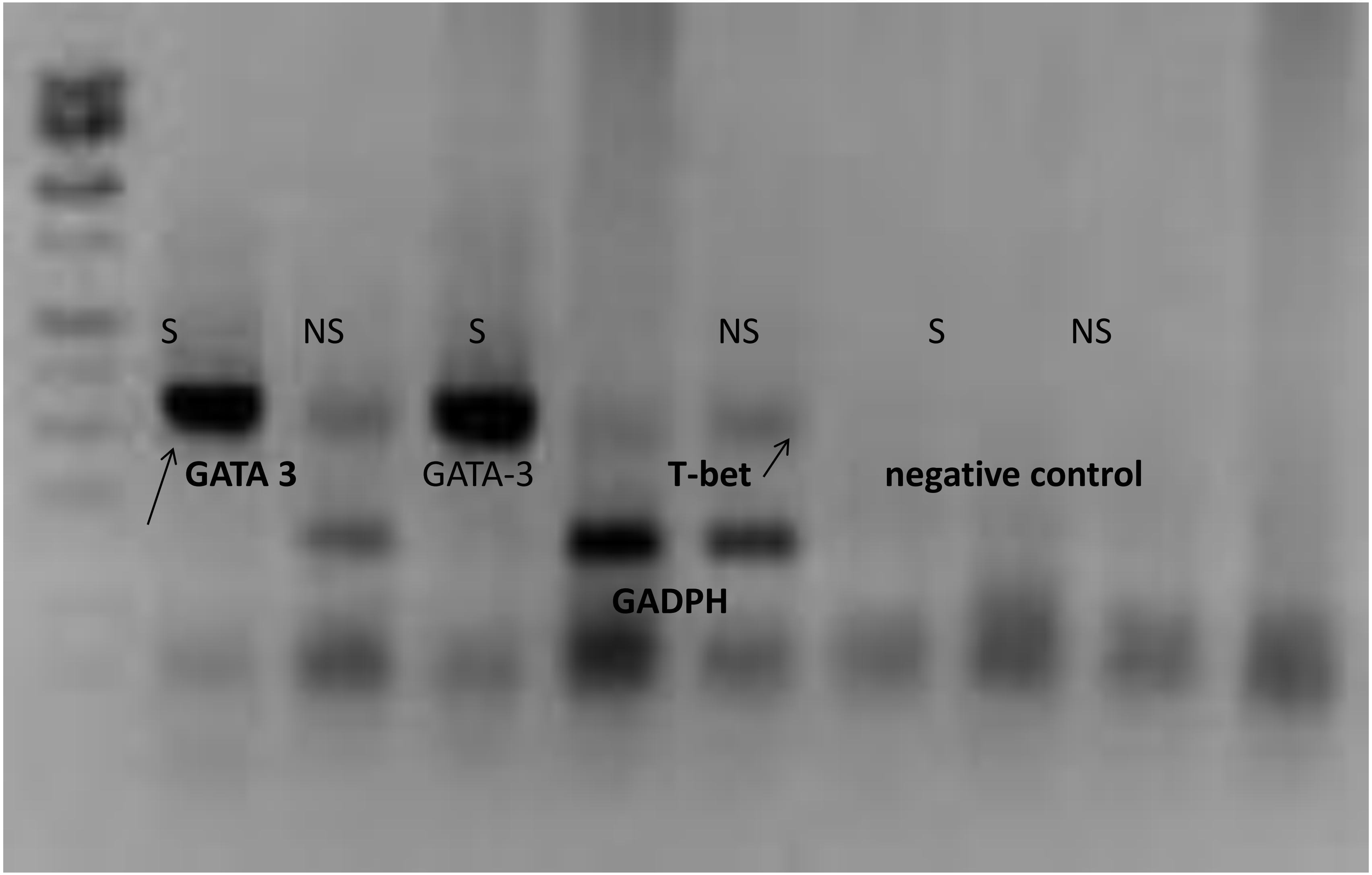

S1 Figure 2. Gel electrophoresis PCR products on gene expression of GATA-3 and T-bet from T cells isolated from genital tracks of *Chlamydia muridarum* infected mice.
