# Supplementary material for: Modulation of T helper 1 and T helper 2 immune balance in a murine stress model during Chlamydia muridarum genital infection
Source: PLoS One. 2020 May 15;15(5):e0226539. doi: 10.1371/journal.pone.0226539 (PMC7228091; doi:10.1371/journal.pone.0226539)
Supplement: S3 Fig — (PDF) [file pone.0226539.s003.pdf]

Figure 8

A

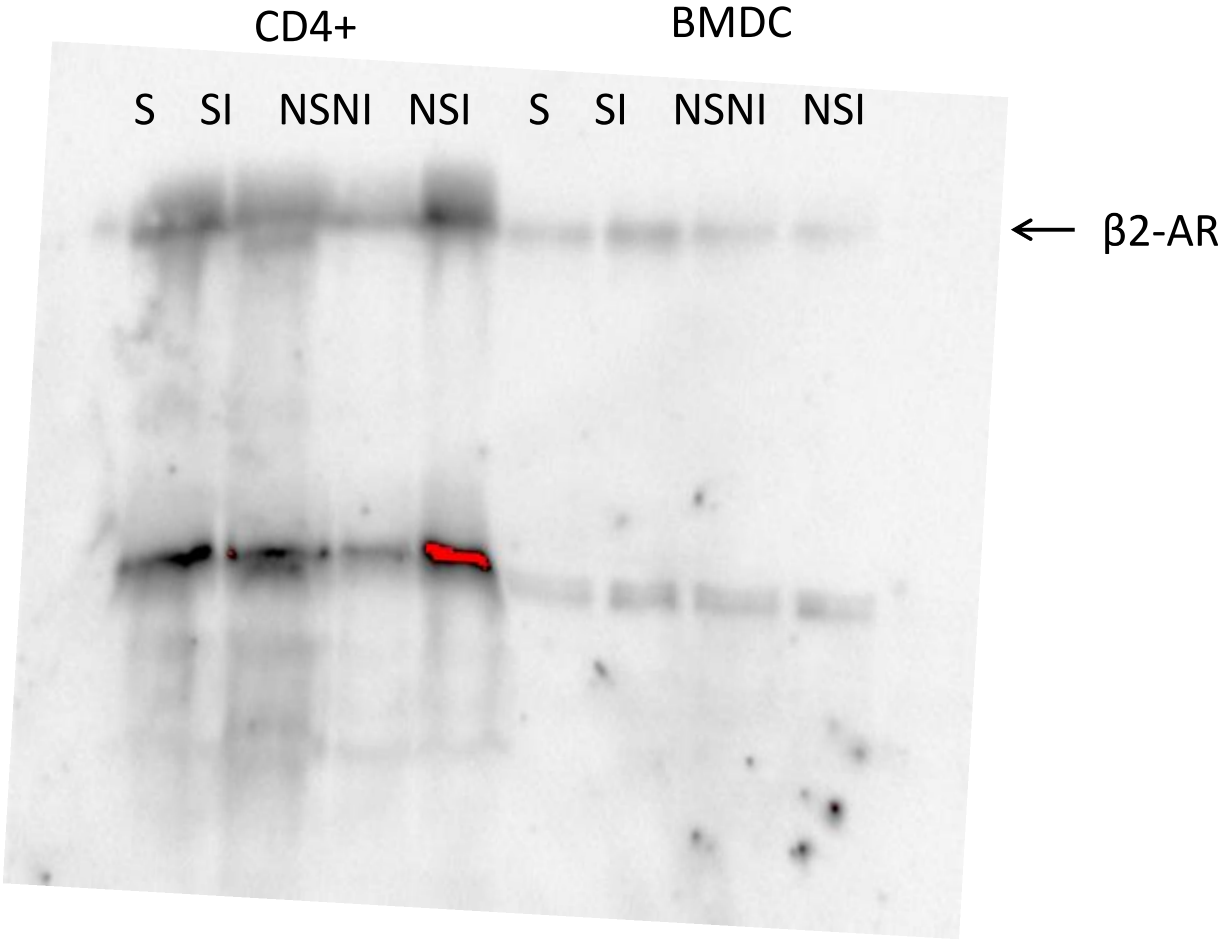

B

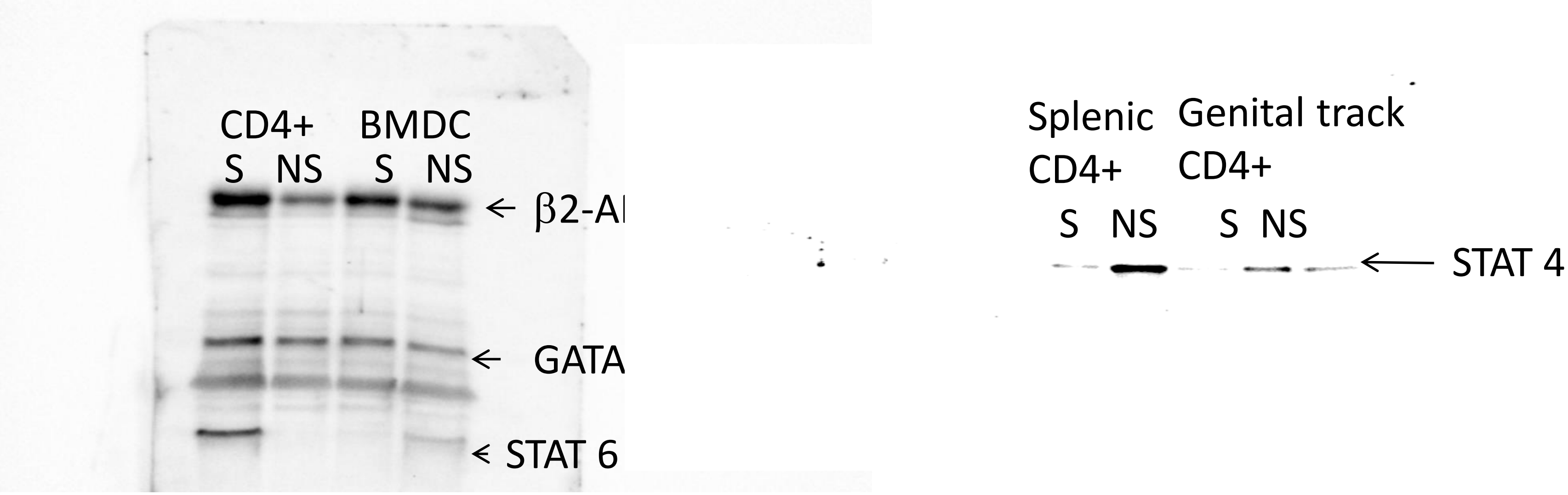

CD4+
BMDC
S NS S NS
β2-A
GATA
STAT 6
Splenic CD4+
Genital track CD4+
S NS S NS
STAT 4

C

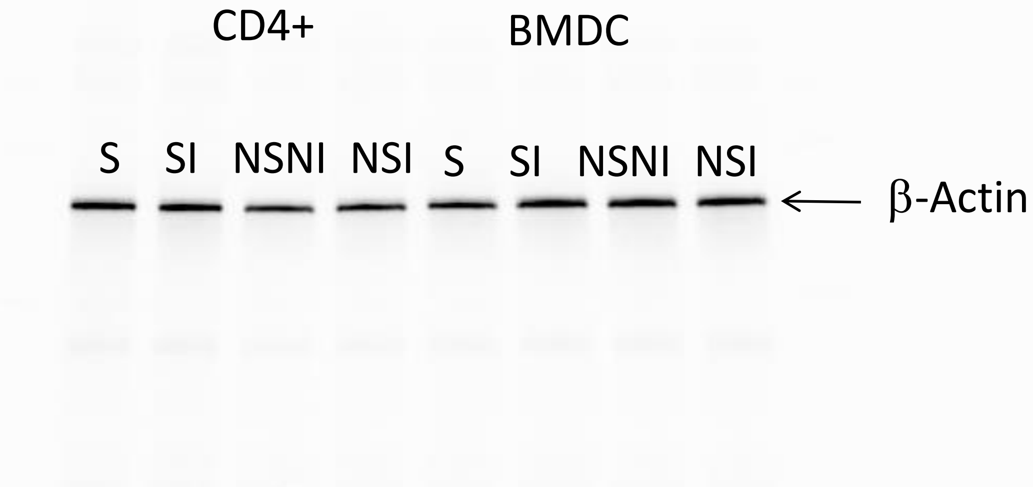

S1 Figure 3: Western blot analysis of transcription factors in CD4 T cells isolated from stressed and non-stressed mice with/without *Chlamydia muridarum* genital infection.
