# Supplementary material for: Modulation of T helper 1 and T helper 2 immune balance in a murine stress model during Chlamydia muridarum genital infection
Source: PLoS One. 2020 May 15;15(5):e0226539. doi: 10.1371/journal.pone.0226539 (PMC7228091; doi:10.1371/journal.pone.0226539)
Supplement: S1 Fig — (PDF) [file pone.0226539.s001.pdf]

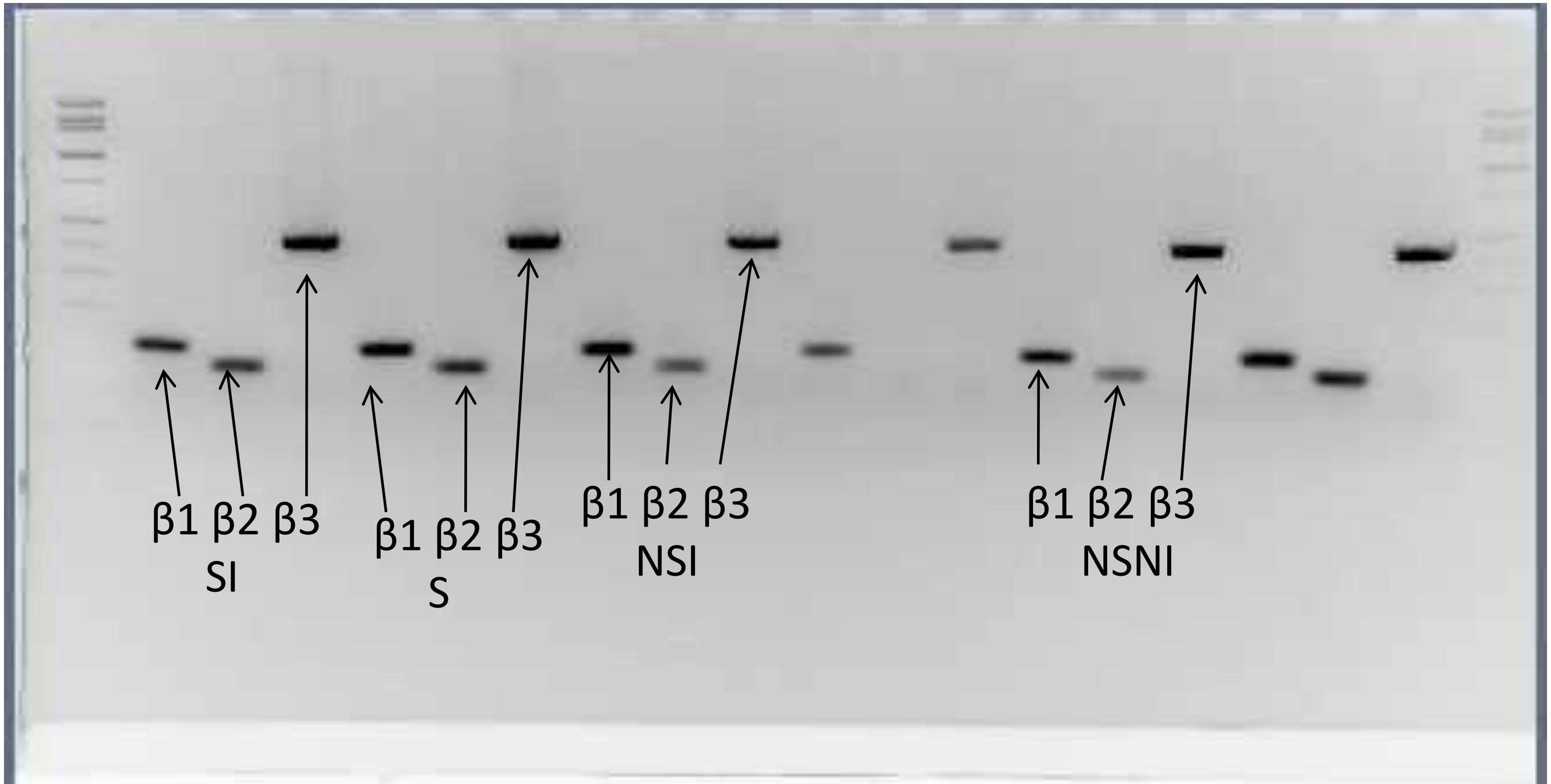

S1 Figure1.Gel electrophoresis profile of beta-1,-2,-3 adrenergic receptors (b-AR) gene expression in CD4+ T cells.
